# Cytoplasmic vacuolation with endoplasmic reticulum stress directs sorafenib induced non-apoptotic cell death in hepatic stellate cells

**DOI:** 10.1038/s41598-021-82381-3

**Published:** 2021-02-04

**Authors:** Sachin Sharma, Shaikh Maryam Ghufran, Sampa Ghose, Subhrajit Biswas

**Affiliations:** 1 Research Laboratory 101, J3 Block, Amity Institute of Molecular Medicine and Stem Cell Research (AIMMSCR), AUUP, Amity University Campus, Sector 125, Noida, Uttar Pradesh 201313 India; 2 Department of Medical Oncology, All India Institute of Medical Sciences, New Delhi, India

**Keywords:** Apoptosis, Liver fibrosis

## Abstract

The activated hepatic stellate cells (HSCs) are the major cells that secrete the ECM proteins and drive the pathogenesis of fibrosis in chronic liver disease. Targeting of HSCs by modulating their activation and proliferation has emerged as a promising approach in the development of anti-fibrotic therapy. Sorafenib, a multi-kinase inhibitor has shown anti-fibrotic properties by inhibiting the survival and proliferation of HSCs. In present study we investigated sorafenib induced cytoplasmic vacuolation mediated decreased cell viability of HSCs in dose and time dependent manner. In this circumstance, sorafenib induces ROS and ER stress in HSCs without involvement of autophagic signals. The protein synthesis inhibitor cycloheximide treatment significantly decreased the sorafenib-induced cytoplasmic vacuolation with increasing cell viability. Antioxidant human serum albumin influences the viability of HSCs by reducing sorafenib induced vacuolation and cell death. However, neither caspase inhibitor Z-VAD-FMK nor autophagy inhibitor chloroquine could rescue the HSCs from sorafenib-induced cytoplasmic vacuolation and cell death. Using TEM and ER organelle tracker, we conclude that the cytoplasmic vacuoles are due to ER dilation. Sorafenib treatment induces calreticulin and GPR78, and activates IRE1α-XBP1s axis of UPR pathway, which eventually trigger the non-apoptotic cell death in HSCs. This study provides a notable mechanistic insight into the ER stress directed non-apoptotic cell death with future directions for the development of efficient anti-fibrotic therapeutic strategies.

## Introduction

Hepatic fibrosis is a wound healing process characterized by the deposition of extracellular matrix (ECM) proteins such as collagen, around the inflamed or injured liver. Excessive deposition of ECM proteins disrupts the normal hepatic architecture and function, resulting in progression to cirrhosis, the major determinant of morbidity and mortality in chronic liver disease patients. The hepatic stellate cells (HSCs) are the principle cells responsible for hepatic fibrosis that become fibrogenic or activated in response to hepatic injury from a quiescent, non-fibrogenic state^1^. Mechanistically, the quiescent state HSCs lose retinoid containing lipid droplets and become activated and transdifferentiate into myofibroblasts. Activated HSCs start to secrete and deposit ECM proteins, which results in fibrotic scar formation in the injured tissue^2,3^. Deactivation or apoptotic clearance of activated HSCs in the fibrotic liver is the key feature for successful fibrosis resolution after the cessation of tissue damage source. Deactivation of fibrogenic response, or clearance of activated HSCs by inducing cell death or apoptosis is a major therapeutic approach in the development of anti-fibrotic therapy^4,5^. Some recent studies have highlighted a few promising anti-fibrotic drugs using apoptotic clearance as therapeutic approaches, sorafenib being one of them^6^. Food and Drug Administration (FDA) approved the multikinase inhibitor Sorafenib as a frontline anti-cancer drug for the treatment of advanced human hepatocellular carcinoma (HCC)^6,7^. Sorafenib attenuates the liver fibrosis by reducing HSC proliferation and inducing cell death. Treatment with sorafenib also induces caspase mediated progressive apoptosis in activated HSCs having shrunken and crescent-shaped nuclear morphology^6^. In another study, it was found that a low dose of sorafenib induces autophagic cell death in activated HSCs, whereas its higher dose inhibits autophagy and induces caspase mediated apoptosis, highlighting the mechanistic interconnections between apoptosis and autophagy^8^. It has been reported that in HCC, sorafenib induces the ER dilation, and activation of the unfolded protein response (UPR) pathway, and these events have direct connection with cytoplasmic vacuolation mediated non-apoptotic cell death^6,9–13^. In present study we found sorafenib induced cytoplasmic vacuolation in HSCs. However, the role of sorafenib induced ER stress, autophagy and their interaction have not been not well documented. While, the cross-talk of ER stress and autophagy are important cellular processes for fibrogenic activity of HSCs^14^, a high demand for extracellular matrix proteins and their folding disturb the ER homeostasis in activated HSCs^14,15^. To maintain ER homeostasis, the ER starts the UPR pathway through three major stress transducers localized at the ER membrane, including activating transcription factor 6 (ATF6), PKR-like ER kinase (PERK), and the inositol requiring enzyme 1α (IRE1α)^16^. When the adaptive UPR response fails to resolve the ER stress, it triggers cell death^17^. A recent study demonstrated that the UPR is an early non-critical event in the activation of HSCs and its prolonged induction triggers ER stress related cell death^18^.

Here we have reported that sorafenib induces non-apoptotic cell death mediated by ER stress which subsequently activates the IRE1α-XBP1s axis of UPR pathway in activated HSCs. Sorafenib also increases reactive oxygen species (ROS) levels in treated cells along with cytoplasmic vacuolation due to ER luminal dilation that are independent of autophagy.

## Results

### Sorafenib induces dose and duration dependent suppression of viability with increased cytoplasmic vacuolation in hepatic stellate cells

**Figure 1:**
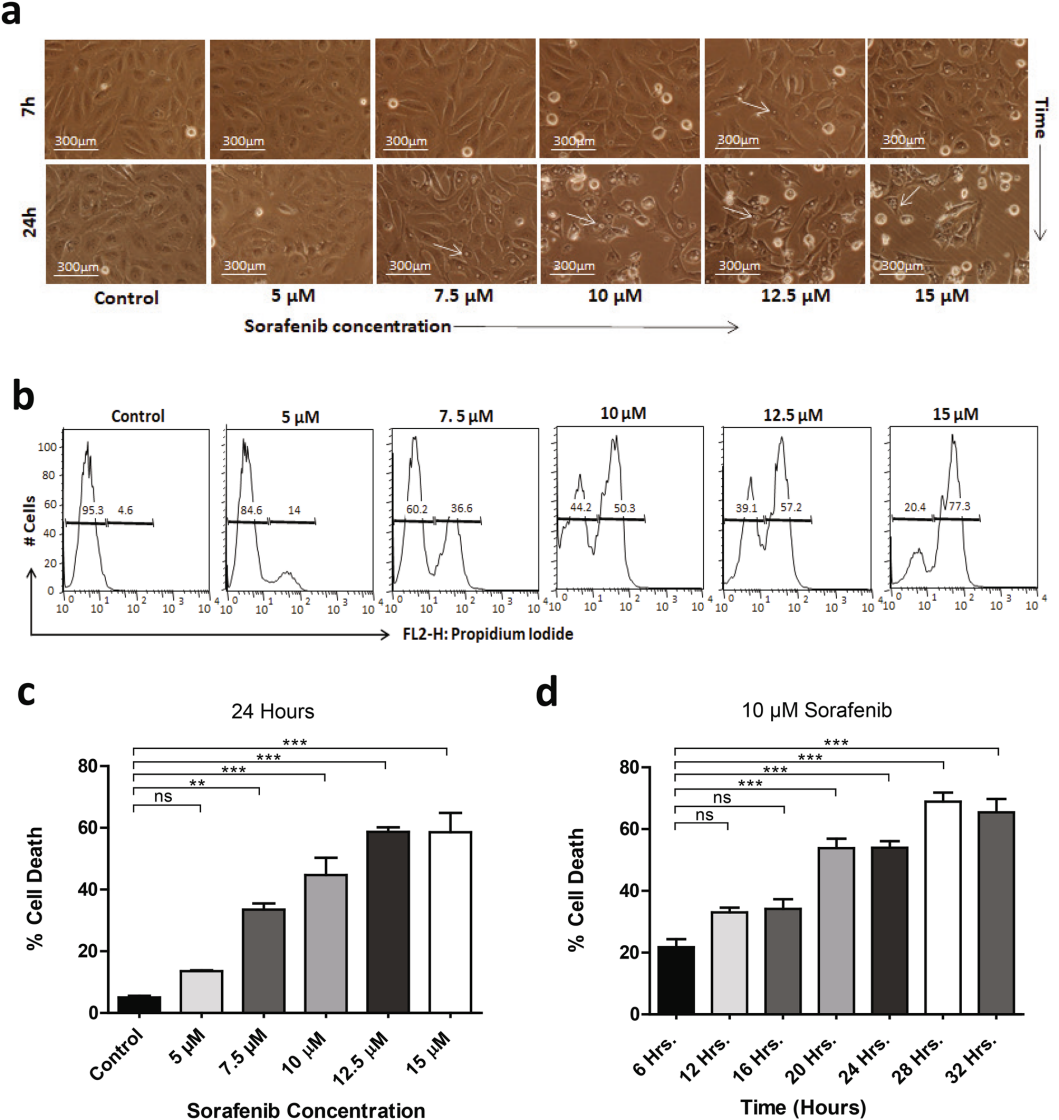
Sorafenib suppress hepatic stellate cell viability and induce cellular death with cytoplasmic vacuolation depending on dose and duration of treatment. (**a**) Phase-contrast microscope showing the LX2 cells treated with different concentration of sorafenib (2.5 µM, 5 µM, 7.5 µM, 10 µM and 15 µM) in 7 h and 24 h in compare with untreated control cells. Cytoplasmic vacuoles were indicated by white arrow. Images were taken using × 20 objective, scale bar: 300 μm. (**b**) Cell death and viability of LX2 cells were shown with propidium iodide (PI) positive and negative populations of LX2 cells after treatment of different concentration of sorafenib for 24 h using flow cytometry. (**c**) The quantification of dose and time dependent percentage (%) cell death of LX2 cells with sorafenib treatment were measured. The bars represent mean ± s.d. from three independent experiments (ns > 0.05, *P < 0.05; **P < 0.01; ***P < 0.001 One-way analysis of variance).

The multikinase inhibitor sorafenib inhibits the fibrogenic activation of HSCs and affects their viability by blocking pro-fibrogenic platelet derived growth factor (PDGF) and transforming growth factor β1 (TGFβ1) receptor mediated signaling^6,19^. To assess the cytotoxicity of sorafenib in activated human HSC cell line, we treated the LX2 cells with different concentrations of sorafenib (5, 7.5, 10, 12.5, and 15 μM) for 24 h. Microscopic analysis showed that sorafenib treatment induces vacuole formation adjacent to the nucleus within the cytoplasm of LX2 cells. Small cytoplasmic vacuoles started to appear when LX2 cells were treated with 7.5 μM dose of sorafenib for 24 h and became bigger in size with increasing concentration of sorafenib (Fig. 1a). Interestingly, the nuclei of HSCs also appeared like a crescent-shape or spherical morphology at 7.5 μM and 10 μM dose of sorafenib as compared to intact nuclei in untreated control cells. Then we measured the viability of LX2 cells through flow cytometry analysis using propidium iodide (PI) staining and found that sorafenib decreased cell viability in a dose dependent manner (Fig. 1b). We further incubated the LX2 cells with a fixed 10 μM dose of sorafenib for various time intervals. The number and size of cytoplasmic vacuoles were increased with increased time duration of sorafenib treatments. These results suggested that the cytoplasmic vacuolation was associated with decreased cell viability and increased duration of treatment (Fig. 1c,d). Here we also compared our results using activated rat hepatic stellate cell line, HSC-T6 where 10 μM dose of sorafenib induced cytoplasmic vacuolation at 24 h similar to activated human HSCs (Suppl. Fig. S1). To eliminate any possibility of cytoplasmic lipid droplet accumulation we performed oil red staining in LX2 cells after treatment with 10 μM dose of sorafenib for 24 h. LX2 cells did not show any accumulated lipid droplets within the cytoplasmic vacuoles (Suppl. Fig. S2a–c). All the above evidences suggest that sorafenib induced cytoplasmic vacuolation and cell death in activated HSCs is dose and time dependent.

### Sorafenib induced cytoplasmic vacuolation in LX2 cells coordinates with non-apoptotic cell death

**Figure 2:**
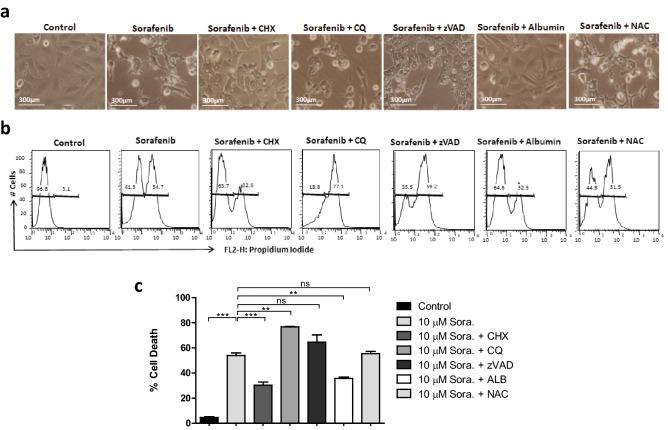
Involvement of non-apoptotic cell death in sorafenib induced LX2 cells depending on dose and time. (**a**) Phase-contrast microscope showing the LX2 cells exposed with 10 µM Sorafenib for 24 h after pre-treatment with protein synthesis inhibitor 25 μM cycloheximide (CHX), autophagy inhibitor, 25 μM chloroquine (CQ), pan-caspase inhibitor, 20 μM Z-VAD-FMK, anti-oxidants 5 μM *N*-acetylcysteine (NAC) and 5 μM humans serum albumin (ALB). Images were taken using × 20 objective, scale bar: 300 μm. (**b**) 10 µM Sorafenib induced percent cell death and viability of LX2 cells were shown with propidium iodide (PI) positive and negative populations of LX2 cells after pre-treatment with above mentioned inhibitors and anti-oxidants using flow cytometry. (**c**) The quantification of sorafenib induced % cell death of LX2 cells with sorafenib (10 µM) treatment after pre-treatment with above mentioned inhibitors were measured in compare with untreated control. The bars represent mean ± s.d. from three independent experiments (ns > 0.05, *P < 0.05; **P < 0.01; ***P < 0.001 One-way analysis of variance).

To investigate the relation between sorafenib induced cytoplasmic vacuolation and cell death in activated HSCs, we pre-treated the LX2 cells with 20 μM caspase inhibitor, Z-VAD-FMK [carbobenzoxy-valyl-alanyl-aspartyl-(*O*-methyl)-fluoromethylketone] 60 min prior to the treatment of 10 μM sorafenib. After 24 h of sorafenib treatment we found that the caspase inhibition was unable to rescue LX2 cells from cell death without alteration of cytoplasmic vacuolation (Fig. 2a,b). These results suggest the involvement of a caspase-independent non-apoptotic cell death in activated HSCs after sorafenib treatment. To confirm the non-apoptotic mode of cell death in sorafenib treated HSCs, we performed DNA fragmentation assay (DNA ladder assay) using agarose gel electrophoresis as the DNA breakdown is an unique feature of apoptotic cell death^20^. In results, no DNA ladder formation was found as an indication of non-apoptotic cell death in 10 μM sorafenib-treated LX2 cells for 24 h (Suppl. Fig. S2d).

To further investigate, whether sorafenib induces autophagy dependent cell death in activated HSCs, we pre-treated LX2 cells with a 25 μM dose of chloroquine (CQ) to inhibit autophagy, prior to treatment of 10 μM sorafenib for 24 h. CQ is an anti-malarial drug that inhibits autophagy by interfering with the fusion of autophagosomes and lysosomes within the cells^21,22^. If sorafenib induced cell death in HSCs occurred via autophagy, the inhibition of autophagy would rescue the cell death and prolong cellular survival in HSCs. Interestingly, CQ unable to prevent cytoplasmic vacuolation and cellular death. In contrast, it enhanced PI + cells (~ 77% in comparison with ~ 54% with only sorafenib treated cells) after exposure of 10 μM sorafenib for 24 h (Fig. 2c). At a higher dose of 15 µM, sorafenib further enhanced the population of PI + LX2 cells pre-treated with CQ to ~ 87% compared to ~ 79% when no pre-treatment was done (Suppl. Fig. S3). These results suggest that autophagy inhibition further enhanced the non-apoptotic cell death in sorafenib treated HSCs without affecting the cytoplasmic vacuole formation.

Several anti-cancer compounds such as Gambogic acid (Xanthonoid), and Cyclosporine A stimulate the cytoplasmic vacuolation associated cell death, and display similar morphological features in the target cells as we observed in our study^10,11^. These compounds triggered the cytoplasmic vacuolation associated cell death mediated with ROS generation and ER stress, which dilate the ER cisternae due to accumulation of misfolded protein in the ER lumen. For further investigation, we pre-treated LX2 cells with 25 μM cycloheximide (CHX), a protein synthesis inhibitor that could reduce the load of protein in ER lumen which may subsequently decrease the ER stress and cell death. Interestingly, here we found that the exposure of CHX reduced both cytoplasmic vacuolation as well as cellular death after treatment with either 10 µM or 15 µM sorafenib for 24 h. This suggests a coordination of protein synthesis regulation with sorafenib induced cytoplasmic vacuolation and caspase independent non-apoptotic cell death (Fig. 2 and Suppl. Fig. S3). The above results provide the clue of the cytoplasmic vacuoles that may emerge through misfolded protein accumulation and ER lumen dilation as a result of sorafenib induced ER stress. To investigate the role of ROS in cytoplasmic vacuolation and cell death, we pre-treated LX2 cells with 5 μM human serum albumin (ALB) or 5 μM *N*-acetylcysteine (NAC) that consist anti-oxidant properties^23,24^. ALB pre-treatment completely inhibited the cytoplasmic vacuole formation in 10 μM sorafenib treated LX2 cells with reduced cell death to ~ 32%. The increased cell viability of sorafenib treated LX2 cells on pre-treatment with ALB was comparable to the increased viability of CHX pre-treated LX2 cells even at high dose of sorafenib (Fig. 2 and Suppl. Fig. S3). However, NAC was unable to protect the LX2 cells from sorafenib mediated cytoplasmic vacuolation and cell death. These observations suggest that sorafenib induced cytoplasmic vacuolation directed cell death in LX2 cells partially depends on ROS generation. Based on these results, we conclude that sorafenib induces cytoplasmic vacuolation with possible induction of ER stress along with caspase independent, non-apoptotic cell death in activated HSCs.

### Alterations of endoplasmic reticulum (ER) are associated with sorafenib induced cytoplasmic vacuolation and non-apoptotic cell death in HSCs

**Figure 3:**
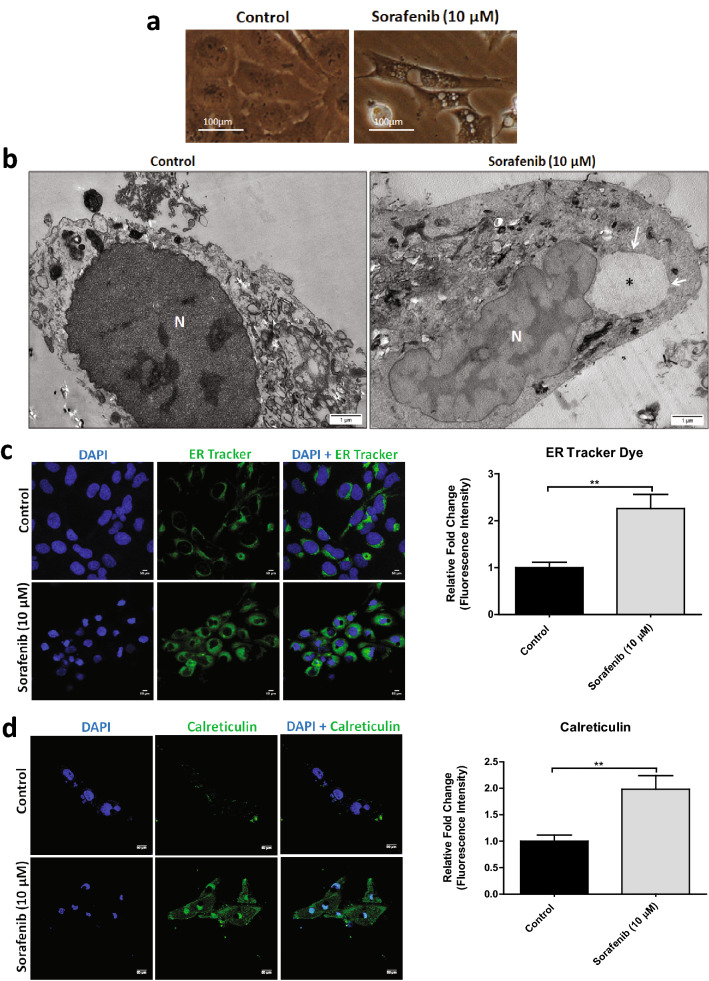
Dose dependent sorafenib induced cytoplasmic vacuolation in LX2 cells are due to ER stress. (**a**,**b**) Phase-contrast and Transmission electron micrograph (TEM) of untreated control and 10 µM sorafenib treated LX2 cells for 24 h. TEM images were taken using × 12,000 magnification, scale bar: 1 μm. Cytoplasmic vacuoles (asterisk, *) were observed in 10 µM sorafenib treated LX2 cells. (**c**) The confocal microscopic images of 10 μM sorafenib treated LX2 cells showed alteration of ER tracker along with cytoplasmic vacuolation with respective to untreated control after 24 h. Nuclei were stained with DAPI (4′,6-diamidino-2-phenylindole). (**d**) The confocal microscopic images showing enhanced calreticulin expression LX2 cells after 10 μM sorafenib treatment after 12 h. Relative fluorescence intensities of ER tracker dye and calreticulin in LX2 cells after exposure with 10 µM sorafenib in compare with untreated control were represented in a graph. The bars represent mean ± s.d. from three independent experiments (ns > 0.05, **P < 0.01 Student's unpaired *t* test).

To investigate whether the sorafenib-induced cytoplasmic vacuolation in activated HSCs through ER dilation, we examined the morphological changes in LX2 cells after the treatment with 10 μM dose of sorafenib for 24 h by Transmission Electron Microscopy (TEM). The untreated control cells showed intact nuclear morphology without dilation of the ER lumen. However, sorafenib treated LX2 cells showed large cytoplasmic vacuoles close to the nucleus (Fig. 3a,b). Sorafenib treated LX2 cells also displayed intact nuclear morphology without chromatin condensation, nuclear fragmentation or plasma membrane blebbing; all features being a hallmark of non-apoptotic cell death^25^. In fact, the vacuoles were surrounded by membranes, some of which were decorated with ribosomes, indicating the chance of intracellular vacuolation from the rough ER (Suppl. Fig. S4). In addition, the vacuoles were surrounded by single layered membrane, and some LX2 cells showed bigger sized vacuoles close to the nucleus arising through ER lumen dilation (Fig. 3b and Suppl. Fig. S4). To further confirm, we stained the ER of both the untreated and 10 μM sorafenib-treated LX2 cells with the ER tracker dye that binds to sulphonylurea receptor of ATP-sensitive K^+^ channel present on the surface of ER. We observed clear cytoplasmic vacuoles close to the nucleus in only sorafenib treated LX2 cells (Fig. 3c). The dilation of the lumen caused the dispersion of the dye throughout the ER. In this context, we also checked the expression of calcium-binding chaperon, calreticulin which is present in the lumen of the ER^26^. 10 μM sorafenib treatment enhanced calreticulin expression after 12 h in both cytoplasm and nucleus (Fig. 3d), suggesting the initiation of aggravated structural disorder of ER and nuclear translocation of calreticulin^26^. Similarly in rat HSC-T6 cells, the calreticulin expression were also elevated in both cytoplasm and nucleus after 10 μM sorafenib treatment at 12 h (Suppl. Fig. S5). These findings confirmed that sorafenib induced cytoplasmic vacuolation mediated non-apoptotic cell death in HSCs are associated with ER dilation.

### Dose dependent influence of sorafenib on LC3 signaling is not associated with cytoplasmic vacuole formation

**Figure 4:**
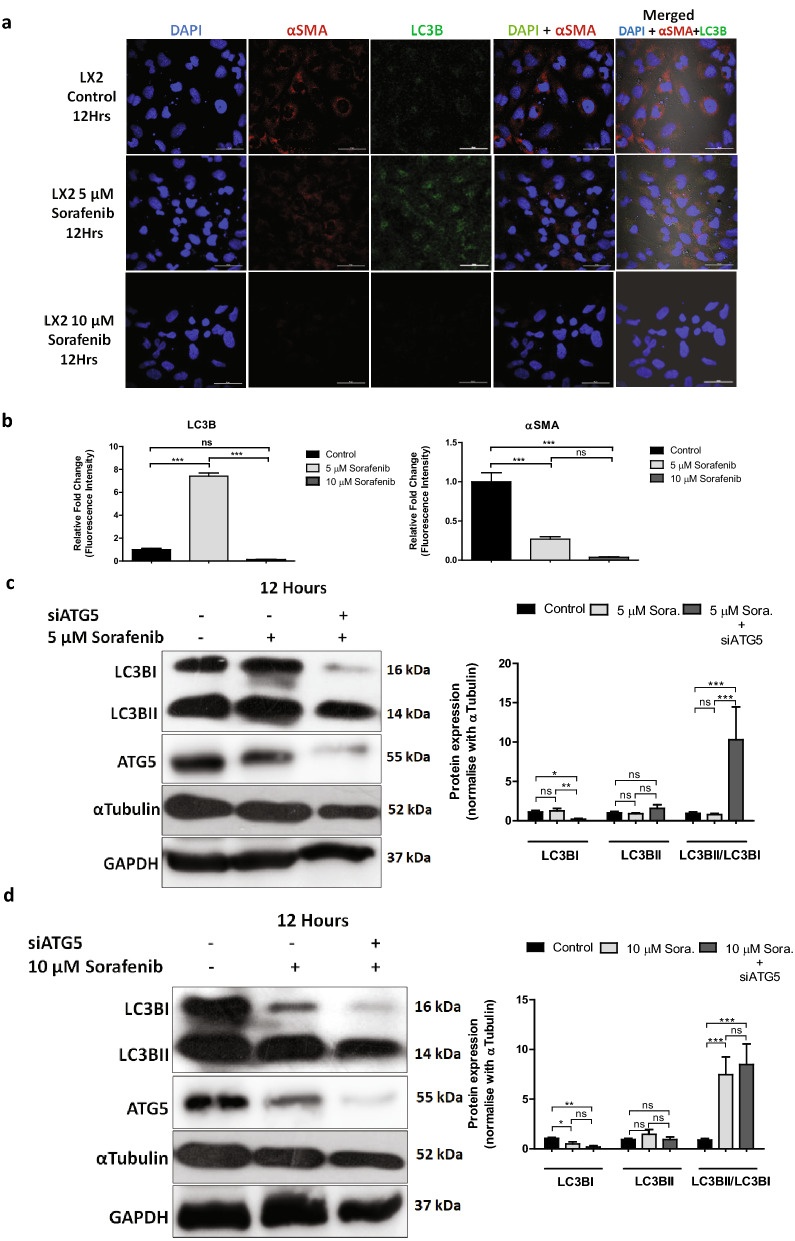
Dose dependent sorafenib induced LC3 signalling. (**a**) The confocal microscopic images showed alteration of LC3B expression in LX2 cells after exposure with low (5 µM) and high (10 µM) concentration of sorafenib for 12 h along with stellate cells activation marker α-SMA. Nuclei were stained with DAPI. Merged pictures showing co-expression of LC3B and α-SMA to evaluate autophagic regulation in activated stellate cells after sorafenib exposure. Images were taken using × 40 objective, scale bar: 50 μm. (**b**) Fluorescence intensity of LC3B and α-SMA quantified and represented as relative fold change with respective to untreated control. The bars represent mean ± s.d. from three independent experiments. (**c**,**d**) Western blot analysis showing protein expression of LC3B conversion in 5 µM and 10 µM sorafenib treated LX2 cells for 12 h. As a negative control the LC3B conversion were also assessed after transfection with 100 nM siRNA against ATG5. Protein level of ATG5 in ATG5 siRNA transfected LX2 cells were shown. Protein expression were quantified using ImageJ software. αTubulin and GAPDH were used as loading controls. Data represent mean ± s.d. from three independent experiments (ns > 0.05, *P < 0.05, **P < 0.01, ***P < 0.001 two-way analysis of variance).

Current literature and our findings based on cytopathological characteristics suggest that the cell death associated with cytoplasmic vacuolation is predominantly due to ER stress and lack of caspase activation^27^. The induction of cytoplasmic vacuolation mediated non-apoptotic and non-autophagic death was reported in several cancers with a mechanism involving ER stress and LC3 (microtubule-associated protein 1 light chain 3)^28^. Alterations in the biochemical nature and subcellular localization of LC3s correlate with autophagy and are used as surrogate markers for its quantification. LC3s (MAP1-LC3A, B, and C) are structural proteins of autophagosomal membranes. While LC3A has been reported to show nuclear and perinuclear localization, LC3B was uniformly distributed throughout the cytoplasm^29^. To investigate the role of autophagy in ER stress and cytoplasmic vacuole formation, we examined the LC3B localization in the cytoplasm of LX2 cells through immunofluorescence (IF) study along with the distribution of hepatic stellate cell activation marker (αSMA) after treatment with low or high dose of sorafenib. At low dose (5 µM) of sorafenib treatment for 12 h, we observed an increased expression of LC3B in LX2 cells with reduced expression of αSMA. Whereas, higher dose (10 µM) of sorafenib for 12 h suppressed the expression of both LC3B and αSMA (Fig. 4a,b). These results suggest that autophagic regulation was not involved with the ER stress mediated cytoplasmic vacuolation, and a higher dose of sorafenib bypasses the requirement of autophagy for inducing cell death in activated HSCs.

To further explain the results, we performed western blotting with low (5 µM) and high (10 µM) dose of sorafenib treated LX2 cells with or without inhibiting ATG5 by siRNA. ATG5 is a critical and indispensable protein for vesicle formation during autophagy^30^. To inhibit autophagy, we inactivated ATG5 in LX2 cells by pre-incubating with 100 nM of ATG5 siRNA prior to sorafenib treatment^31^. Then we examined the alteration of autophagic flux in 5 µM sorafenib treated LX2 cells after inactivation of ATG5 compared to wild type ATG5 sorafenib treated LX2 cells. We observed a similar ratio of LC3BI to LC3BII in control cells with respective to 5 µM sorafenib treated cells, possibly due to delayed autophagosome turn over that accumulated and enhanced LC3BII expression (Fig. 4c). Similarly we observed some autophagic vacuoles in the TEM images of untreated LX2 cells (Suppl. Fig. S4), may be to maintain the cellular homeostasis because autophagy also plays a vital role in fibrogenic responses of activated HSCs^32^. In contrast, when LX2 cells were treated with 10 µM dose of sorafenib for 12 h, the LC3BI expression was reduced, which was comparable to the expression of LC3BI in LX2 cells treated with a lower dose of sorafenib (5 µM) following ATG5 inactivation (Fig. 4d). Together with the above findings, we conclude that a high dose of sorafenib (10 µM) inhibits autophagy and mediates non-autophagic cell death in activated HSCs. These results were also in concordance with our previous findings with autophagic inhibitors, where we showed that CQ was unable to suppress the cytoplasmic vacuolation and non-apoptotic cell death induced by 10 µM sorafenib in LX2 cells (Fig. 2).

### ROS is critically involved in sorafenib induced ER stress but cannot alone influence the cytoplasmic vacuolation mediated cell death

**Figure 5:**
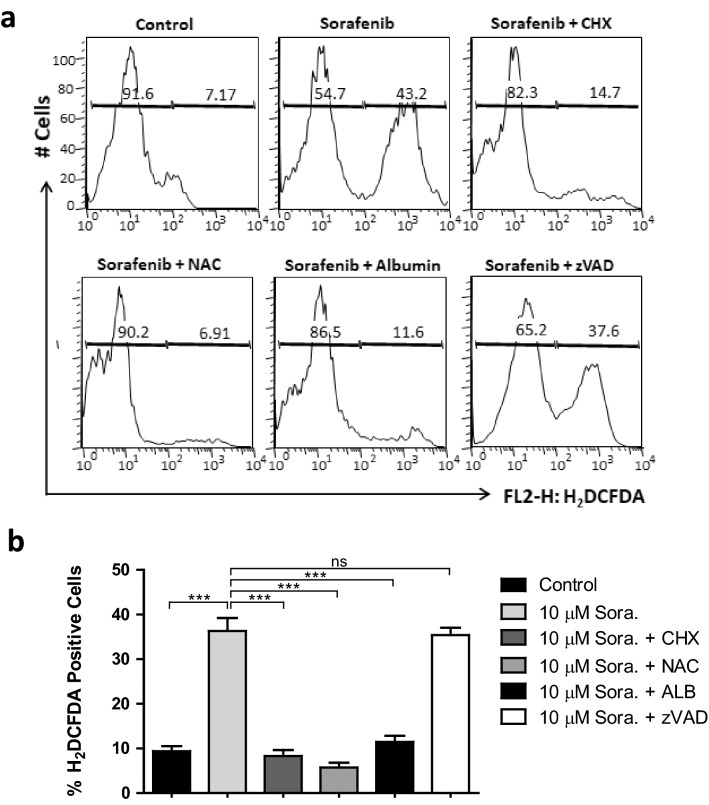
ROS participate in sorafenib induced stress. (**a**) Sorafenib induced cellular reactive oxygen species (ROS) were measured by positive population of the fluorogenic dye, 2′,7′-dichlorodihydrofluorescein diacetate (H_2_DCFDA) in LX2 cells with or without pre-treatment of different inhibitors and anti-oxidants *N*-acetylcysteine (NAC), human serum albumin (ALB), protein synthesis inhibitor cycloheximide (CHX), and pan-caspase inhibitor vZAD. (**b**) The quantification of percentage (%) H_2_DCFDA positive LX2 cells with sorafenib exposure after pre-treatment with above mentioned inhibitors. Statistical analysis performed with grouped data to show the significant difference among relative H_2_DCFDA positive cells. Data represent mean ± s.d. from three independent experiments (ns > 0.05, ***P < 0.001 One-way analysis of variance).

Various studies have reported that ROS-mediated ER stress play a critical role in sorafenib induced cell death in various cancer types^33,34^. Based on these findings, we predicted that ROS-mediated ER stress can play a critical role in sorafenib induced cytoplasmic vacuolation and cell death in activated HSCs. Therefore, we analysed the intracellular ROS that labeled with 2′,7′-dichlorodihydrofluorescein diacetate (H_2_DCFDA) fluorescent signals and quantified by flow cytometry analysis. We observed that the production of ROS was significantly increased in LX2 cells after treatment with 10 µM sorafenib for 24 h (Fig. 5). To determine whether the increased intracellular ROS levels mediate the sorafenib induced ER stress in LX2 cells, we inhibited ROS production by pre-treating LX2 cells with anti-oxidants NAC and ALB prior to 10 μM dose of sorafenib treatment. ALB and NAC inhibited the sorafenib induced ROS generation as indicated by a decrease in H_2_DCFDA positive cell population to ~ 11% and ~ 6% respectively compared to ~ 36% H2DCFDA positive cells in LX2 cells treated with sorafenib alone. As mentioned above the NAC pre-treated LX2 cells showed no change in cytoplasmic vacuolation and cell death after sorafenib treatment (Fig. 2). On the other hand, ALB pre-treatment reduced both the sorafenib induced ROS production, cytoplasmic vacuolation, and cell death in LX2 cells after 10 µM of sorafenib treatment for 24 h (Figs. 2 and 5). These observations suggest the indirect involvement of ROS in sorafenib induced cytoplasmic vacuolation mediated cell death. Interestingly, CHX pre-treatment also resulted a similar ROS suppression effect as observed with ALB, however CHX was unable to completely rescue vacuole formation as seen with ALB pre-treated HSCs. LX2 cells pre-treated with caspase inhibitor vZAD-FMK showed no alteration in sorafenib induced ROS production, cytoplasmic vacuolation as well as cell death (Figs. 2 and 5). Here, we conclude that sorafenib mediated ROS generation induced ER dilation that subsequently results in cytoplasmic vacuolation and triggers non-apoptotic cell death in activated HSCs.

### Sorafenib induces non-apoptotic cell death in activated HSCs through ROS, ER stress, and UPR pathway

**Figure 6:**
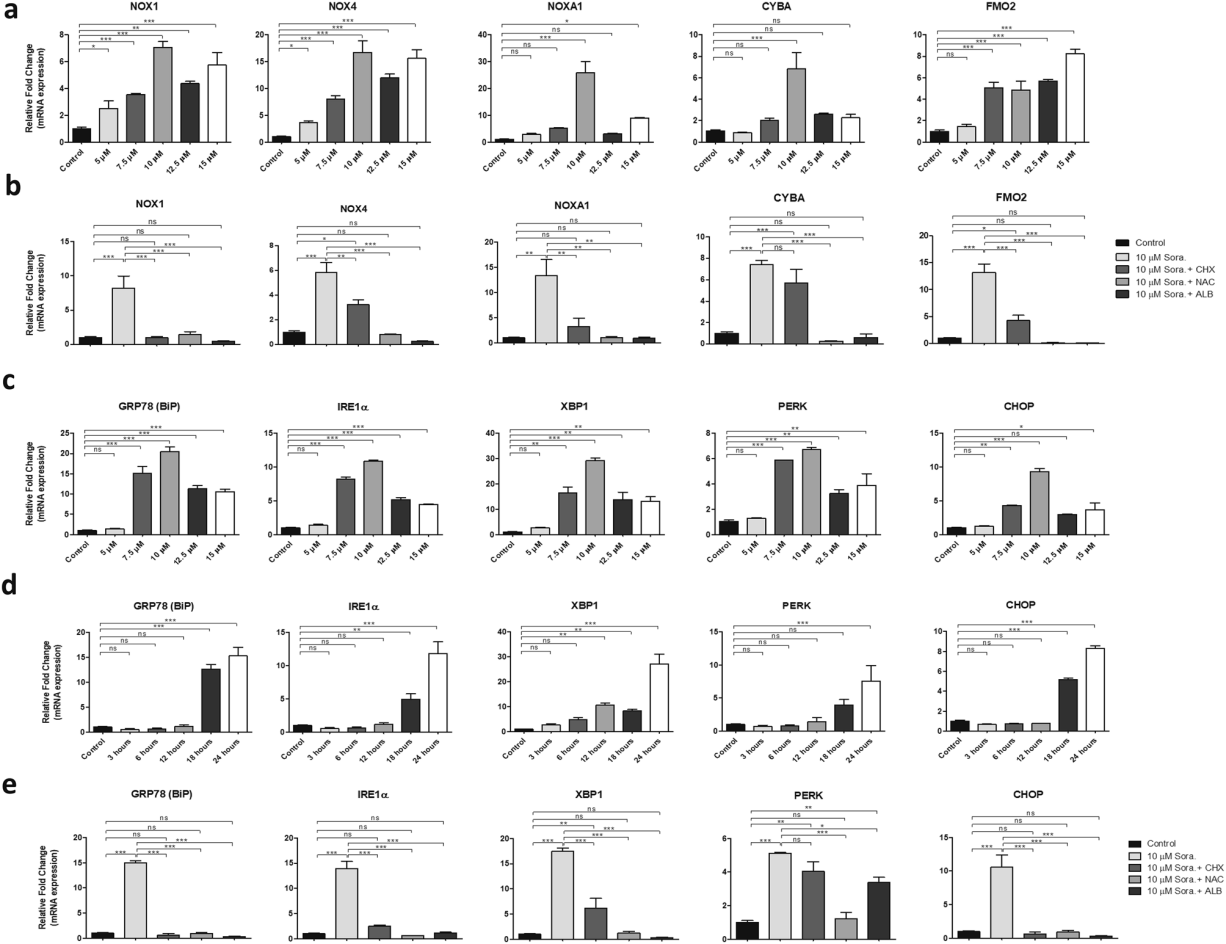
Influence of Sorafenib on ROS and ER stress signalling. (**a**) qPCR showing different concentration of sorafenib induced fold changes of relative mRNA expressions of ROS generating enzyme gene such as NOX1, NOX4, NOXA1, CYBA, FMO2 for 24 h. (**b**) The relative mRNA expression of 10 µM sorafenib induced ROS generating enzyme genes after 24 h without and with pre-treatment of CHX, NAC and albumin (ALB). (**c**) qPCR showing different concentration of sorafenib induced fold changes of relative mRNA expression of ER stress or unfolded protein response (UPR) markers such as GPR78 (BiP), IRE1α, PERK, XBP1 and CHOP after 24 h. (**d**) Time dependent relative mRNA expression of 10 µM sorafenib induced ER stress markers were shown in different concentration and different time point up to 24 h. (**e**) The relative mRNA expression of 10 µM sorafenib induced ER stress markers without and with pre-treatment of CHX, NAC and albumin. Fold changes are normalised with expression of 18S rRNA. Data represent mean ± s.d. from three independent experiments (ns > 0.05, *P < 0.05, **P < 0.01, ***P < 0.001 One-way analysis of variance).

Excessive ROS production can generate oxidative stress which further triggers the ER stress. It may lead to accumulation of large amounts of unfolded or misfolded proteins in the ER lumen and initiate the cellular ER stress response known as un-coupled protein response (UPR) pathway^35^. In this context, we evaluated the gene expression of ROS generating enzymes such as nicotinamide adenine dinucleotide phosphate (NADPH) oxidase 1 (NOX1), NADP oxidase 4 (NOX4), NADPH Oxidase Activator 1 (NOXA1), Cytochrome B-245 Alpha Chain (CYBA), and Flavin-containing monooxygenase 2 (FMO2). We found that sorafenib induced oxidative stress in LX2 cells by upregulating the mRNA expression of NOX1, NOX4, NOXA1, CYBA, and FMO2 in a dose dependent manner. LX2 cells showed maximum expression of these genes when treated with 10 μM sorafenib dose for 24 h (Fig. 6a). Pre-treatment with CHX, NAC, and ALB suppressed the increased mRNA expression of ROS generating enzymes in LX2 cells after 10 µM sorafenib treatment for 24 h (Fig. 6b). These results suggest the involvement of ROS signals in sorafenib induced cytoplasmic vacuolation and cell death. However, the anti-oxidant NAC was unable to supress the sorafenib induced cytoplasmic vacuolation mediated non-apoptotic cell death.

To investigate the possible ER stress mediated signaling, we quantified the mRNA expression of ER stress or UPR pathway markers such as Binding immunoglobulin protein/(GPR78/BiP), inositol-requiring enzyme 1 (IRE1α), PKR-like ER kinase (PERK), X-box-binding protein 1 (XBP1), and C/EBP Homologous Protein (CHOP) in LX2 cells on treatment with different concentration of sorafenib and different time durations with 10 μM sorafenib. We found that sorafenib enhanced the mRNA expression of UPR markers in a dose and time dependent manner. LX2 cells treated with the 10 μM sorafenib dose for 24 h showed the highest expression of UPR genes (Fig. 6c,d). However, the sorafenib induced upregulation of UPR genes in LX2 cells was attenuated on pre-treatment with CHX, NAC and ALB (Fig. 6e).

Upon ER stress, IRE1/endoRNAse activity regulates the expression of the transcription factor cleaved XBP1 (XBP1s)^36^. Here the mRNA level of XBP1 showed a steady increase significantly from 12 h time point after 10 μM sorafenib treatment, determining a possible involvement of IRE1α-XBP1s axis to induce ER stress.

### Dose dependent sorafenib induced UPR is associated with functional activation of the IRE1α-XBP1s axis

**Figure 7:**
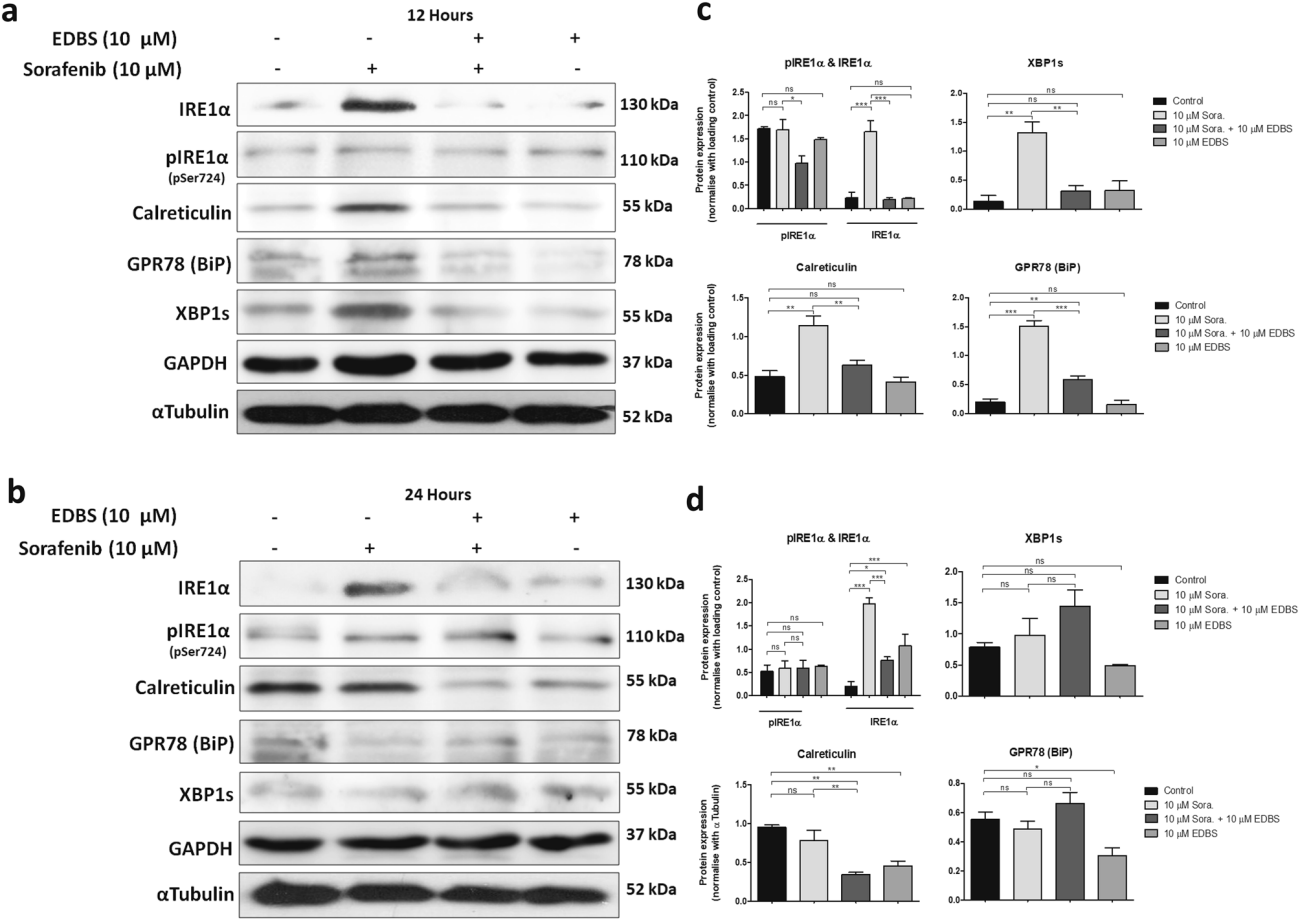
Higher concentration of sorafenib induced UPR pathway associated with IRE1α-XBP1s axis. (**a**,**b**) Western blot analysis showing IRE1α, pIRE1α, GRP78 (BiP), calreticulin and XBP1s expression after treatment with 10 µM sorafenib treatment for 12 h and 24 h. αTubulin and GAPDH were used as a loading control. The immunoblots were depicted the influence of inhibitor of IRE1α 3-Ethoxy-5,6-dibromosalicylaldehyde (EDBS) on 10 µM sorafenib induced protein level of IRE1α, pIRE1α, GRP78 (BiP), calreticulin and XBP1s. (**c**,**d**) Protein expression level were quantified using ImageJ software. GAPDH and αTubulin were used as a loading control. Relative protein ratios (normalized with loading control) were shown in a plot graph. Data represent mean ± s.d. from three independent experiments (ns > 0.05, *P < 0.05; **P < 0.01, ***P < 0.001 One-way analysis of variance).

To further confirm the role of the IRE1α-XBP1s axis of the UPR to ER stress, we analysed the protein expression patterns of related genes through western blot. It was found that the IRE1α were significantly overexpressed in LX2 cells on treatment with 10 μM sorafenib at both the 12 h and 24 h time points (Fig. 7a–d). During ER stress IRE1α activates the endoribonuclease domain, which primarily acts through XBP1^37^. Here the protein levels of XBP1s were also enhanced in LX2 cells after treatment with 10 μM sorafenib for 12 h. Calreticulin and GRP78 (BiP) chaperon proteins that bind to misfolded or un-folded proteins were also upregulated after sorafenib treatment for 12 h (Fig. 7a,c). As per literature IRE1α autophosphorylation enhanced further oligomerization of the protein to stimulate RNase activity^38^. In contrast, sometimes IRE1α can bypass its autophosphorylation to cleave XBP1 for activation of the UPR pathway^36^. In our results we found that phosphorylation of IRE1α (pIRE1α) were unaltered in LX2 cells without or with 10 μM sorafenib at both 12 and 24 h of treatment.

To further explain the effect of ER stress on the cytoplasmic vacuolation-mediated cell death, we blocked the ER stress signalling using 3-Ethoxy 5, 6-dibromosalicylaldehyde (EDBS). EDBS is a non-competitive reversible inhibitor that binds specifically to the IRE1α protein to inactivate its endoribonuclease activity^39^. To inhibit the IRE1α protein we pre-treated the LX2 cells with 10 μM EDBS for 60 min prior to exposure of sorafenib for 12 h and 24 h. EDBS pre-treatment decreased the expression of the GRP78, IRE1α, XBP1s and calreticulin after 12 h of 10 μM sorafenib treatment (Fig. 7a,c). There was a very marginal reduction of basal level pIRE1α in EDBS pre-treated LX2 cells after sorafenib treatment for 12 h. We again confirmed the overexpression of IRE1α and its suppression through pre-treated EDBS after 10 μM sorafenib treatment in rat HSC-T6 cells for both 12 h and 24 h (Suppl. Fig. S6), suggesting the involvement of IRE1α mediated UPR pathway in sorafenib induced ER stress.

**Figure 8:**
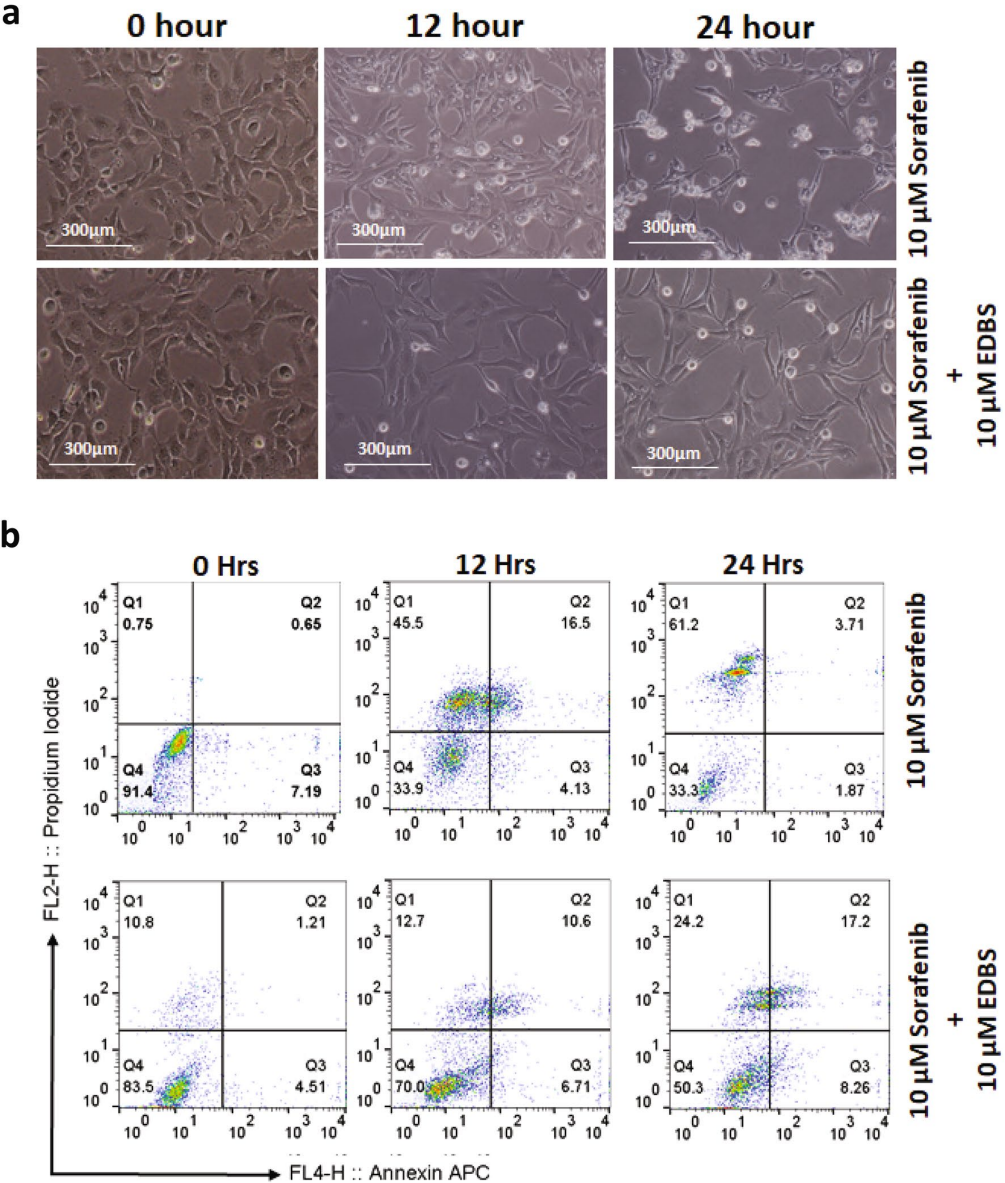
Inhibition of IRE1α through EDBS pre-treatment delayed sorafenib mediated cytoplasmic vacuolation and cell death. (**a**) Phase-contrast microscope showing the effect of pre-treated LX2 cells with EDBS on sorafenib (10 µM) induced cytoplasmic vacuolation at 12 h and 24 h. (**b**) The effect of EDBS on 10 µM sorafenib induced cell death at 12 h and 24 h were determined by Annexin V staining with propidium iodide exclusion using flow cytometry analysis. PI positive population were estimated to measure percent cell death.

Most strikingly, when we pre-treated human HSCs, LX2 cells and rat HSC-T6 with EDBS there was significant reduction in cytoplasmic vacuolation after 12 h of 10 μM sorafenib treatment, whereas when sorafenib treatment was extended to 24 h, the vacuolation was delayed (Fig. 8a and Suppl. Fig. S7). Here, we also found that EDBS not only effectively suppressed the cytoplasmic vacuolation but also drastically reduced the population of PI + LX2 cells after sorafenib treatment at both the 12 h and 24 h time points (Fig. 8b) These results demonstrate the induction of ER stress with the involvement of IRE1α-XBP1s axis during sorafenib induced cytoplasmic vacuolation-mediated non-apoptotic cell death in activated HSCs.

## Discussion

As the central effector of liver fibrosis, activated HSCs have been the focus of many studies examining mechanisms underlying the disease. The conception of activated HSCs as a target for the treatment of liver fibrosis has stimulated the investigation of pathways that promote HSC apoptosis, as a means to facilitate disease regression^40,41^. Various anti-fibrotic agents have been identified such as gliotoxin, sulfasalazine, and tectorigenin that target the activated HSC cell survival and proliferation, ultimately inducing cell death to limit the fibrogenic activity of HSCs^42–44^. Sorafenib has also been reported to have anti-fibrotic effects by limiting cell proliferation and inducing apoptosis in activated HSCs that leads to fibrosis regression^6^. Originally, sorafenib is a frontline anti-cancer drug that is used for treatment of advance HCC. Mechanistically, sorafenib blocks vascular endothelial growth factor (VEGF) and platelet derived growth factor (PDGF) receptors to suppress tumour angiogenesis or inhibit MAP Kinase pathways to suppress tumour cell proliferation^45,46^. The suppression of proliferation and induction of apoptosis are accompanied by a down regulation of cyclins and cyclin dependent kinases (Cdks)^6^. Jiang et al. showed that HSCs treated with sorafenib exhibited shrunken chromatin that was aggregated and condensed inside the nuclear membrane, with crescent-shaped or spherical nuclear morphology^6^. Other studies have reported that sorafenib reduced proliferation, induced autophagy and apoptosis in HSCs^8^. Sorafenib induced autophagy and apoptosis in HSCs have been shown to interlink through mechanisms of cross-talk^47^. In our study, we highlighted a new mechanism of ER stress induced autophagy independent non-apoptotic cell death in activated HSCs after the treatment with sorafenib.

We found that sorafenib induces cytoplasmic vacuolation adjacent to the nucleus in activated HSCs and subsequently cell death. Sorafenib induced vacuolations become bigger with increased dose and time duration of treatment. However, pre-treatment with CHX, rescued the activated HSCs from the sorafenib induced effects. CHX treatment halts the synthesis of proteins and their subsequent accumulation in the ER lumen eventually rescues the HSCs from ER stress. Based on these results we reasoned that cytoplasmic vacuolation after the sorafenib treatment are dilated ER cisternae. TEM analysis and confocal analysis of sorafenib treated HSCs with ER tracker dye and calreticulin expression further confirmed the results. Interestingly, we found a basal autophagic flux in activated human HSCs and LX2 cells by TEM analysis and confirmed with LC3B protein expression through immunoblotting and confocal microscopy. As autophagy is essential for cellular homeostasis, some basal autophagy is present in activated HSCs. Moreover autophagy is a critical event for the induction of fibrogenic response. It is rapidly up-regulated as an adaptive response under a variety of cellular stress conditions including nutrient deprivation, oxidative stress, and infections^32^.

**Figure 9:**
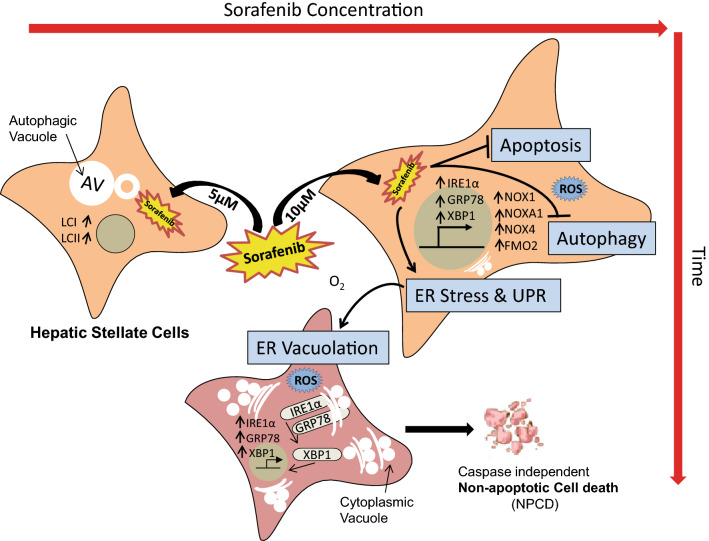
Diagram showing dose dependent sorafenib induced cellular death through ER vacuolation. Therapeutic treatment with sorafenib leads to deactivation of HSCs by means of overlapping cellular processes such as autophagy, apoptosis, and non-apoptotic death. Depending on concentration and duration of sorafenib treatment the activated stellate cells can undergo autophagy. On the other hand, higher concentration of sorafenib lead activated stellate cells to undergo cytoplasmic vacuole mediated non-apoptotic cell death by suppressing apoptotic and autophagic pathway but with increasing reactive oxygen species and ER stress. ER vacuolation were mediated by functional activation of UPR pathway involving GPR78, IRE1α and XBP1s.

Friedman et al. showed the induction of autophagy in hepatic stellate cells in Carbon tetrachloride (CCl_4_) and Thioacetamide (TAA) induced liver injury model^48^. They suggested that autophagy fulfills the high energy demand required to initiate and maintain the stellate cell activation by liberation of free fatty acid (FFA), lipid droplet (LD) mobilization, and mitochondrial-oxidation. Autophagy is also involved in a cell death process called as autophagic cell death that differs from apoptosis in the presence of characteristic autophagosomes and autophagolysosomes within the dying cells^22,49^. Consistent with previous studies, we found that a low concentration of sorafenib for short duration of treatment induces autophagy, however at high concentrations and longer durations of sorafenib treatment inhibits autophagy. Interestingly, CQ treatment did not rescue the HSCs from the cytoplasmic vacuolation mediated cell death. The vacuoles start to appear only after treating activated HSCs with a higher dose of sorafenib i.e. 7.5 µM for 24 h. This indicated that the cytoplasmic vacuoles are not autophagic vacuoles, and a higher dose of sorafenib treatment bypasses autophagic cell death to cytoplasmic vacuolation mediated cell death. Studies also reported that cell death processes switched from autophagic to apoptotic and non-apoptotic depending upon whether the exposure to stimuli was extended to longer durations or the drug concentration was increased. Many studies have already demonstrated that sorafenib inhibits the proliferative activity of activated HSCs through caspase mediated apoptosis^50,51^. Surprisingly, when we pre-treated LX2 cells with caspase inhibitor before the sorafenib treatment, we found that caspase inhibition was unable to rescue from their cell death without effecting vacuole formation. During flow cytometry analysis with annexin-PI, we found lesser number of early apoptotic and late apoptotic LX2 cells at 12 h or 24 h of 10 μM sorafenib treatment. Thus, previous findings together with our current results confirm that the viability of LX2 cells decreases due to cytoplasmic vacuolation mediated non apoptotic cell death upon sorafenib treatment depending on the dose and time (Fig. 9). The cytoplasmic vacuole formation in HSCs at higher dose of sorafenib treatment is caspase and autophagy independent.

The role of ER stress has been studied in a variety of diseases including liver fibrosis^52^. Kim’s group demonstrated the effects of ER stress on the activation of HSCs^53^. Similar to other reports we have highlighted the sorafenib induced ROS mediated ER stress and the accumulation of misfolded proteins in the ER lumen resulting in the ER dilation^12^. To overcome this stress or to restore the normal ER function, the ER starts the UPR pathway to avert the ER stress or induce cell death if stimuli persists^54,55^. In our study, we have shown that dose dependent influence of sorafenib induces the gene expression markers of ER stress such as XBP1, CHOP, GPR78 (BiP), IRE1α, PERK, and oxidative stress markers such as NOX1, NOX4 NOXA1, CYBA, and FMO2. However, CHX, anti-oxidant NAC, and ALB pre-treated HSCs showed decreased expression of both ER stress and oxidative stress markers after sorafenib treatment. We observed that ALB completely abolished the cytoplasmic vacuole formation and rescued the HSCs from sorafenib induced cell death but we could not find similar results with using NAC. XBP1 showed a steady increase in mRNA expression with sorafenib treatment, further indicating the involvement of the IRE1α-XBP1s axis of the UPR. We evaluated also the expression of pIRE1α in sorafenib treated HSCs and compared with IRE1α through immunoblot analysis. The expression of pIRE1α was not significantly enhanced after either treatment of sorafenib as compared with control. EDBS showed no effect and was unable to decrease the pIRE1α expression either with or without sorafenib treatment in HSCs. On the other hand, EDBS inhibited the overexpressed IRE1α, GRP78 and XBP1s which eventually enhanced the cell viability in sorafenib treated HSCs indicating sorafenib induced cytoplasmic vacuolation through the IRE1α-XBP1s UPR axis.

Thus, the present study delivers unique insights into the anti-fibrotic effects of sorafenib treatment in activated HSCs, and highlights the complex interplay between ER stress and cell death pathways. This study provides evidence for a new mechanism of sorafenib action in activated HSCs within the liver fibrosis microenvironment. Further investigation of the molecular mechanisms underlying sorafenib induced cytoplasmic vacuolation mediated non-apoptotic cell death may lead to the development of a novel therapeutic approach for the more effective management of liver fibrosis.

## Methods

### Chemical reagent

Reagents used in the present study were as follows: Sorafenib Tosylate (475207-59-1; Santa Cruz Biotechnology, Santa Cruz, California, USA); Caspase family inhibitor Z-VAD-FMK (1010-100; BioVision, CA USA); Cycloheximide (CHX), a protein synthesis inhibitor (C1988; Sigma-Aldrich, St. Louis, Missouri, USA); antioxidant *N*-acetyl-cysteine (NAC) (Santa Cruz Biotechnology, Santa Cruz, California, USA); Human serum albumin (ALB) (A1653; Sigma-Aldrich, St. Louis, Missouri, USA), an autophagy inhibitor Chloroquine (CQ) (H0915; Sigma-Aldrich, St. Louis, Missouri, USA), 2′,7′-dichlorodihydrofluorescein diacetate (H2DCFDA) (D399; Invitrogen, San Diego, CA, USA), 3-Ethoxy-5,6-dibromosalicylaldehyde (EDBS) (SML0149; Sigma-Aldrich, St. Louis, Missouri, USA), Antifade Mounting Medium with DAPI (H-1200; Vector Laboratories, San Diego, CA), Propidium Iodide (PI) (P4170; Sigma-Aldrich, St. Louis, Missouri, USA) and dimethyl sulfoxide (DMSO) (D2650; Sigma-Aldrich St. Louis, Missouri, USA).

### Cell culture

Human hepatic stellate cell line LX-2, obtained from Dr. Scott L. Friedman, and rat hepatic stellates cell line HSC-T6 purchased from Elabscience (EP-CL-0116; Elabscience, Houston, Texas, USA), routinely cultured in Dulbecco's modified Eagle medium (DMEM) high glucose supplemented with 2% (v/v) and 10% (v/v) fetal bovine serum (FBS), respectively, with 4 mM l-glutamine, 100 IU/ml Penicillin/100 µg/ml Streptomycin at 37 °C with 5% CO_2_ in humidified atmosphere. We usually passage cells with trypsin/EDTA, and performed experiments after the cell achieve 70–80% confluency. All the experiments were conduct in serum free routine cell culture media without prior serum deprivation. To examine the effect of inhibitors on sorafenib treatment, we pre-treated the cells with inhibitors 60 min prior to treatment of sorafenib.

### Measurement of cell viability and % cell death

0.5 × 10^6^ cells were seeded in 60 mm cell culture dish and cultured overnight for the attachment. Next, cells were washed with cell culture grade 1X phosphate buffered saline (PBS) buffer (TS1101, Himedia, India) before the treatment to remove residual FBS. Respective cells treatment was performed in serum free DMEM high glucose containing 4 mM l-glutamine, 100 IU/ml penicillin/100 μg/ml streptomycin. Untreated cells were consider as untreated control in experiments. Cell viability assay was performed using propidium iodide (PI) (P4170; Sigma-Aldrich, St. Louis, Missouri, USA), flow cytometric analysis. Samples from different groups were collected by trypsinization, and washed twice with cold PBS buffer. Cells were re-suspended in 100 µl PBS and added PI with final concentration of 1 µg/ml and incubate at 2–8 °C for 5 min in dark. Cell analysis was performed on BD FACS Calibur flow cytometer (BD Biosciences). All experiments were performed in triplicate.

### Annexin-propidium iodide (PI) flow cytometry

Untreated or treated cells were detached using trypsin/EDTA and wash with 1 × PBS buffer to remove cellular debris. Cell pellets were collected through centrifuge at 600×*g* for 5 min at 4 °C. Cells pellet were then re-suspended into 100 μl 1 × annexin binding buffer and incubated with 5 μL of FITC-conjugated Annexin V (556419; BD Biosciences, USA) for 30 min. After incubation, wash the cells with 1 × annexin binding buffer two times through centrifuge at 600×*g* for 5 min each at 4 °C. Cells pellet were re-suspended in 200 µl 1 × annexin binding buffer and added PI with final concentration of 1 µg/ml and incubate at 2–8 °C for 5 min in dark. Cell analysis was performed on BD FACS Calibur flow cytometer (BD Biosciences) within 1 h. All experiments were performed in triplicate.

### Transmission electron microscopy

For TEM analysis, 2 × 10^6^ cells were seeded in 100 mm cell culture dish and treated with 10 μM Sorafenib for 24 h in serum free DMEM high glucose containing 4 mM l-glutamine, 100 IU/ml penicillin/100 μg/ml streptomycin. After treatment, we washed the cells to remove cell debris and trypsinized to collect cell pellet. Next, cells pellets were prefixed in 2% paraformaldehyde, 2% glutaraldehyde, (0.1 M sodium phosphate buffer, pH 7.4) for overnight hours at 4 °C and washed with PBS buffer. Post-fixing was carried out in 1% osmium tetroxide and 1.5% potassium ferrocyanide for 1 h. After dehydration with 50–100% alcohol, the cells were embedded in Poly/Bed 812 resin (Pelco), polymerized, and observed under Transmission electron microscope at Advanced Technology Platform Centre (ATPC), Regional Centre for Biotechnology, Haryana, India.

### Confocal microscopy with ER tracker and Immunofluorescence of treated cells

0.15 × 10^6^ cells were cultured on coverslip (2850-18; Corning, New York, USA), overnight cell for attachment. After the attachment, cells were treated with 10 μM Sorafenib dose for 24 h. Next after the treatment cell were washed with 1 × PBS to remove cell debris. For ER tracker dye staining, cells were incubated with 100 nM ER Tracker dye (E34251; Invitrogen, San Diego, CA, USA) for 30 min at 37 °C/5%CO_2_. Next, Cells were washed with 1 × PBS buffer and fixed in 4% paraformaldehyde (PFA) for 10 min. After fixation cells were washed with 1 × PBS three times and glass coverslip mounted on glass slide (2947-75; Corning, New York, USA) with antifade mounting medium with DAPI. For immunofluorescence, cells were fixed with 4% paraformaldehyde (PFA) after the sorafenib treatment for 10 min and washed with 1 × PBS three times. Next, cells were incubated with blocking reagent (5% bovine serum albumin, 0.3% Tritron X-100 in PBS) for 60 min for blocking. Antibodies were diluted in antibody dilution buffer (1% bovine serum albumin, 0.15% Tritron X-100 in PBS). Next, after the blocking, to determine the LC3B and α-SMA expression in LX2 cells, we incubated with primary antibody LC3B and α-SMA along with respective fluorescence-tagged secondary antibody mentioned in Supplementary informations. Then, cover clip was mounted with VECTASHIELD antifade mounting medium with DAPI (#H-1200). Confocal images were taken using Advanced Nikon A1 confocal microscope at Amity University Uttar Pradesh, India and at Advanced Technology Platform Centre (ATPC), Regional Centre for Biotechnology, Haryana, India.

### Small-interfering RNA (siRNA) transfection

Small-interfering RNA (siRNA) against *ATG5* and non-specific scrambled siRNA were purchased from Dharmacon. LX2 cells were cultured in 6 well plates. Lipofectamine 2000 (11668-027; Invitrogen, San Diego, CA, USA) was mixed with serum free DMEM containing 100 nM siRNA or scrabbled siRNA final concentration and incubated for 20 min at room temperature. Transfection mixture were incubated on cells at 37 °C in 5% CO2 for 6 h in serum free routine cell culture media. Experiments were performed after the 3 days of siRNA transfection.

### Measurement of reactive oxygen species (ROS)

To measure intracellular ROS production, 0.5 × 10^6^ cells were seeded in 60 mm cell culture dish. We performed the experiments after over-night attachment of cells. We detached the cells with trypsin/EDTA and washed with 1 × PBS buffer to remove cellular debris. We incubated cells with 5 µM 2′,7′-dichlorodihydrofluorescein diacetate (H_2_DCFDA) for 30 min in the dark, washed with 1 × PBS buffer and further processed for flow cytometry analysis using BD FACS Calibur flow cytometer (BD Biosciences). All experiments were performed in triplicate. Data were analysed using FlowJo software (BD Biosciences).

### RNA isolation and quantitative polymerase chain reaction (qPCR)

Total RNA was isolated from treated and untreated cells using Trizol reagent (Invitrogen, San Diego, CA, USA), and cDNA was prepared using random primers and reverse transcriptase (K1631; Thermo Fisher Scientific, Waltham, MA, USA). We used following primers for real time XBP, CHOP GPR78 (BIP), IRE1, PERK, NOX1, NOX4, NOXA1 CYBA, FMO2and 18S ribosomal RNA. Details of primer sequence mentioned in supplementary table (Suppl. Table S1). Real-time PCR was performed using Powerup SYBR green master mix (A25742 Applied Biosystems, Waltham, MA, USA). The copy number of the target mRNA in each sample was normalized as a ratio using the copy number for 18S rRNA in the denominator.

### Western blot analyses

Treated or untreated cells were washed with PBS and homogenized in RIPA lysis buffer in presence of 1 × protease inhibitor (11697498001; Roche, St. Louis, Missouri, USA) and 1 × phosphatase inhibitor (4906845001; Roche, St. Louis, Missouri, USA). Protein concentrations were determined using Bradford protein assay (20279; Thermo Fisher Scientific, Waltham, MA, USA). 30 µg protein lysates were separated by 12% (w/v) SDS-PAGE, and proteins were transferred to PVDF membrane (1620177; BioRad, CA, USA). Membrane were incubated with primary antibody for overnight at 4 °C with gently shaking. Secondary ant-rabbit or anti-mouse was incubated for 2 h and visualized using an Enhanced Chemiluminescence (ECL) detection kit (34094; Thermo Fisher Scientific, Waltham, MA, USA). Details of primary and secondary antibodies mentioned in Supplementary Table (Suppl. Table 2). For statistical analyses and densitometry analyses was measured using prism and ImageJ software.

### Statistical analysis

All data were presented as mean ± SD (standard deviation) from at least three separate experiments. Student’s *t* test was applied to evaluate the differences between treated and control groups. Data from multiple groups were analyzed by one-way or two way ANOVA using Prism-GraphPad. For all the tests, the level of significance was values of *P* < 0.05.

## Supplementary Information

Supplementary Information.
