# Supplementary material for: Cytoplasmic vacuolation with endoplasmic reticulum stress directs sorafenib induced non-apoptotic cell death in hepatic stellate cells
Source: Sci Rep. 2021 Feb 4;11:3089. doi: 10.1038/s41598-021-82381-3 (PMC7862314; doi:10.1038/s41598-021-82381-3)
Supplement: Supplementary file 1 — Supplementary Information. [file 41598_2021_82381_MOESM1_ESM.pdf]

Supplementary Informations

Title: Cytoplasmic vacuolation with endoplasmic reticulum stress directs sorafenib induced non-apoptotic cell death in hepatic stellate cells.

Authors: Sachin Sharma[1], Shaikh Maryam Ghufran[1], Sampa Ghose[2], Subhrajit Biswas[1*]

CONTENTS

1. Supplementary Materials and Methods
2. Supplementary Figure Legends
3. Supplementary Tables
4. Supplementary Figures

Supplementary Materials

Methods

Cell culture

Rat hepatic stellates cell line HSC-T6 purchased from Elabscience (EP-CL-0116; Elabscience, Houston, Texas, USA), routinely cultured in Dulbecco's modified Eagle medium (DMEM) high glucose supplemented with 10% (v/v) fetal bovine serum (FBS), respectively, with 4 mM L-glutamine, 100 IU/ml Penicillin/100 µg/ml Streptomycin at 37^0C with 5% CO2 in humidified atmosphere. We usually passage cells and performed experiments at 70-80% confluency. All the experiments were conduct in serum free routine cell culture media without prior serum deprivation. In experiment, we pre-treated cells with inhibitors 60 minutes prior to treatment of sorafenib.

Immunofluorescence of treated cells

$0.15X10^6$ HSC-T6 cells were cultured on coverslip (2850-18; Corning, New York, USA), overnight cell for attachment. After the attachment, cells were treated with 10 µM Sorafenib dose for 24 hours. Next after the treatment cell, wash with PBS followed by fixation with 4% paraformaldehyde (PFA). Next, cells were incubated with blocking reagent and incubated with primary and secondary antibodies (Supplementary table 2). VECTASHIELD antifade mounting medium with DAPI (#H-1200).

Oil Red Staining

$0.15X10^6$ LX2 cells were seeded in 60 mm dish for overnight cell attachment. After the cell attachment, cells were treated with 100 µM palmitic acid (BSA-conjugated) for 48 hours. Next, after the treatment cell, wash with PBS followed by fixation with 4% paraformaldehyde (PFA). Washed the cells with 1X PBS and incubated with 0.2% Oil Red (dissolved in isopropanol) for 10 minutes. Washed with distil water and counter stained with haematoxylin for nuclear stain.

Agarose gel DNA fragmentation assay

$0.5X10^6$ LX2 cells were seeded in 60 mm dish for overnight cell attachment. Cell death was induced with treatment of 10 µM sorafenib for 24 hours. TNFα (20 ng/ml) along with CHX (50 µg/ml) treatment for 24 hours were used to induce DNA fragmentation as positive control of apoptotic cell death [1]. Next, cells were lysed with detergent buffer (10 mM Tris (pH 7.4), 5 mM EDTA, 0.2% Triton) and incubate for 60 minutes in ice. Centrifuge at 27,000 x g for 30 min and collected supernatant. Added 5 M NaCl ice-cold, ethanol and 3 M sodium-acetate (pH 5.2) to

participate DNA. Next, centrifuge 20,000 x g for 20 minutes, discard supernatants and redissolve pellet in extraction buffer (10 mM Tris and 5 mM EDTA). Added RNase (10 mg/ml) and proteinase K (20 mg/ml) incubated 5 hours and followed by overnight at 65°C. Extracted DNA with phenol/chloroform/isoamyl alcohol (25:24:1) and wash with ethanol followed by air dry. DNA pellet was resuspended in Tris-acetate EDTA buffer. Genomic DNA was visualized in 1.8% agarose gel with 1 µg/ml ethidium iodide through ImageQuant LAS500 gel imaging system (GE Healthcare).

Western Blot

Treated or untreated control HSC-T6 cells were washed with PBS and homogenized in RIPA lysis buffer in presence of 1X protease inhibitor (11697498001; Roche, St. Louis, Missouri, USA) and 1X phosphatase inhibitor (4906845001; Roche, St. Louis, Missouri, USA). Protein concentrations were determined using Bradford BSA protein assay (20279; Thermo Fisher Scientific, Waltham, MA, USA). 30 µg protein lysates were separated by 12% (w/v) SDS-PAGE, and proteins were transferred to PVDF membrane (1620177; BioRad, CA, USA). Membrane were incubated with primary antibody and secondary ant-rabbit or anti-mouse antibody, details mentioned in supplementary Table ((Supplementary table 2). Membrane were visualized using an enhanced chemiluminescence (ECL) detection kit (34094; Thermo Fisher Scientific, Waltham, MA, USA). For statistical analyses and densitometry analyses was measured using prism and ImageJ software

1 Novo, E. *et al.* Overexpression of Bcl-2 by activated human hepatic stellate cells: resistance to apoptosis as a mechanism of progressive hepatic fibrogenesis in humans. *Gut* **55**, 1174-1182, doi:10.1136/gut.2005.082701 (2006).

Supplementary Figure Legends

Supplementary Figure S1. **Sorafenib induces cytoplasmic vacuolation in rat hepatic stellate cell HSC-T6 cell line.** Rat HSC cell line HSC-T6 cells were treated with 10 μM sorafenib for 12 hours and 24 hours. Cytoplasmic vacuoles were detected by phase-contrast microscopy (indicated by white arrow). Images were taken using 20X objective, scale bar: 100 μm.

Supplementary Figure S2. **Sorafenib induced no lipid droplets as cytoplasmic vacuoles in activated HSC LX2 cells. a** Positive control oil red stain LX2 cells, LX2 cells were incubation with 100 μM palmitic acid (BSA- palmitate conjugate) for 48 hours. **b, c** Oli red stained after the treatment with 10 μM sorafenib for 24 hours without and with haematoxylin. Images were taken using 10X objective. **d** DNA ladder assay were performed with 10 μM sorafenib treated LX2 cells for 24 hours. TNFα (20 ng/ml) along with CHX (50 ng/ml) were used to induce DNA fragmentation as positive control in DNA ladder assay.

Supplementary Figure S3. **Protein synthesis inhibition rescued, but autophagy inhibition failed to rescue from higher dose sorafenib (15 μM) induced cell death in activated HSC LX2 cells. a** 15 μM Sorafenib induced cell death and viability of LX2 cells were shown with propidium iodide (PI) positive and negative populations in LX2 cells after pre-treatment with protein synthesis inhibitor 25 μM cycloheximide (CHX) and autophagy inhibitor, 25 μM chloroquine (CQ) using flow cytometry **b** Quantification of 15 μM sorafenib induced percentage (%) of cell death in treated LX2 cells were measured. The bars represent mean ± s.d. from three independent experiments. (*$P<0.05$; **$P<0.01$; ***$P<0.001$ One-way analysis of variance).

Supplementary Figure S4. **Sorafenib induces ER dilation as cytoplasmic vacuoles in activated HSC LX2 cells. (**a) TEM image of untreated control LX2 cells taken using 15,000X magnification, scale bar: 1 μm. Cells nuclear envelope (NE) appears intact, ribosome bound ER (rER). (b) Higher magnification (40,000X) cells of selected area (white square area) shown basal level of autophagosome-lipid bilayer with organelle (AP) and lipid bilayer autophagic vacuole (AV), scale bar: 200 nm. (c) TEM image of LX2 cells treated with 10 μM sorafenib for 24 hours. No plasma membrane blebbing and chromatin degradation observed. Image taken using

12,000X magnification, scale bar: 1 μm. (d) Higher magnification (50,000X) cells of selected area (white square area) shown mitochondria (M), smooth ER (ER), cytoplasmic vacuole (asterisk, *), dilated ER (white arrow), scale bar: 200 nm.

Supplementary Figure S5. **Sorafenib increases calreticulin expression in rat hepatic stellate cell HSC-T6 cells. a** Confocal microscopic image of HSC-T6 cells treated with 10 μM sorafenib for 12 hours with respective to untreated control HSC-T6 cells. Images were taken using 63X oil immersion objective, scale bar: 50 μm. **b** Relative fold change of fluorescence intensity quantification of HSC-T6 cells treated with 10 μM sorafenib for 12 hours with respective to untreated control HSC-T6 cells. The bars represent mean ± s.d. from three independent experiments. (**P<0.01 Student's Unpaired t-test).

Supplementary Figure S6. **Sorafenib induces IRE1α in rat hepatic stellate cell HSC-T6 cells.** Western blot showing IRE1α expression in 10μM sorafenib treated HSC-T6 cells for 12 hours (**a, b)** and 24 hours (**c, d)** with or without pre-treatment of 10 μM EDBS. Protein expression level of IRE1α were quantified using ImageJ software. GAPDH were used as a loading control. Relative protein ratios (normalized with GAPDH) were used to quantify fold change relative to control (no treatment) were shown in a plot graph. Data represent mean ± s.d. from three independent experiments. (*P<0.05; **P<0.01; ***P<0.001 One-way analysis of variance).

Supplementary Figure S7. **LX2 treated with 10 μM EDBS appears morphologically similar to LX2 untreated control. a** Phase-contrast microscopic image of LX2 cells, treated with 10 μM EDBS for 12 hours and 24 hours with or without pre-treatment of IRE1α inhibitor 10 μM EDBS for 12 hours and 24 hours with respective untreated LX2 control. Images were taken using 10X objective, scale bar: 100 μm. **b** Phase-contrast microscopic image of HSC-T6 cells, treated with 10 μM sorafenib for 12 hours and 24 hours with or without pre-treatment of 10 μM IRE1α inhibitor, EDBS. Images were taken using 20X objective, scale bar: 100 μm

Supplementary Table 1: Primer nucleotide Sequences

Gene Symbol	Nucleotide Sequence **(5' to 3')**	
	**Forward Primer (FP)**	**Reverse Primer (RP)**
CYBA	AGATCGGAGGCACCATCA	GGTTGACCTGGGGACCTC
FMO2	TGATGATGTCCCAAGTCGTC	TGTTCTCCTCCACTGTTCCA
NOX1	TCTTTGTTAATTGCCCCTCAA	CCTGCTGCTCGGATATGAAT
NOX4	CAGTCTTAACCGAACCAGCTCT	GGTAAACTCTGCCGGTTTTG
NOXA1	ATCTTCCCCAAGTGCTTCGT	GCATCATGGACACAGCATCA
GRP78	GCCGTCCTATGTCGCCTTC	TGGCGTCAAAGACCGTGTTC
PERK	ACGATGAGACAGAGTTGCGAC	AATCCCACTGCTTTTTACCATGA
IRE1α	CTGTATCTTGGGCGAACAGAAT	TGCCAGACATAAAAGGCCACC
CHOP	TGGAAGCCTGGTATGAGGAC	TGTGACCTCTGCTGGTTCTG
XBP1	AAACAGAGTAGCAGCTCAGACTGC	TCCTTCTGGGTAGACCTCTGGGAG
18S	GCAATTATTCCCCATGAATG	GGCCTCACTAAACCATCCAA

Supplementary Table 2: Primary and secondary antibodies details

**Target Protein**	**Company**	**Application**	**Dilution**
LC3B	CST, #3868	Immunoblot	1:1000
		Immunofluorescence	1:200
ATG5	CST, #12994	Immunoblot	1:1000
αSMA	Abcam, #ab7817	Immunofluorescence	6.82 µg/ml
Calreticulin	CST, #12238	Immunoblot	1:1000
		Immunofluorescence	1:200
IRE1α	CST, #3294	Immunoblot	1:1000
pIRE1 α [p Ser724]	NOVUS, # NB100-2323	Immunoblot	1:1000
GPR78 (BiP)	CST, #3177	Immunoblot	1:1000
XBP1s	CST, #12782	Immunoblot	1:1000
αTubulin	CST, #2144	Immunoblot	1:2000
GAPDH	Sigma-Aldrich, G8795	Immunoblot	1:10,000
Anti-rabbit HRP-conjugated secondary antibody	Jackson Immuno Research, #111-035-144	Immunoblot	1:10,000
Anti-mouse HRP-conjugated secondary antibody	Jackson Immuno Research, #115-035-003	Immunoblot	1:10,000
Anti-rabbit FITC-conjugated secondary antibody	Abcam, #ab6717	Immunofluorescence	1:250
Anti-mouse Alexa Fluor 647-conjugated secondary antibody	Abcam, #ab150115	Immunofluorescence	1:500

Suppl. Fig. S1

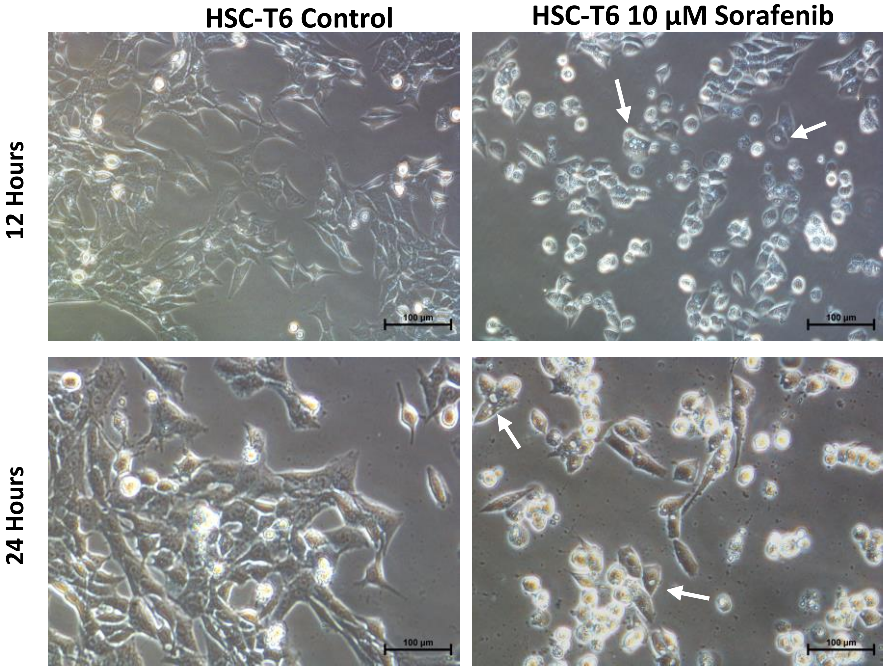
HSC-T6 Control
HSC-T6 10 µM Sorafenib
12 Hours
24 Hours
100 µm
100 µm
100 µm
100 µm

Suppl. Fig. S2

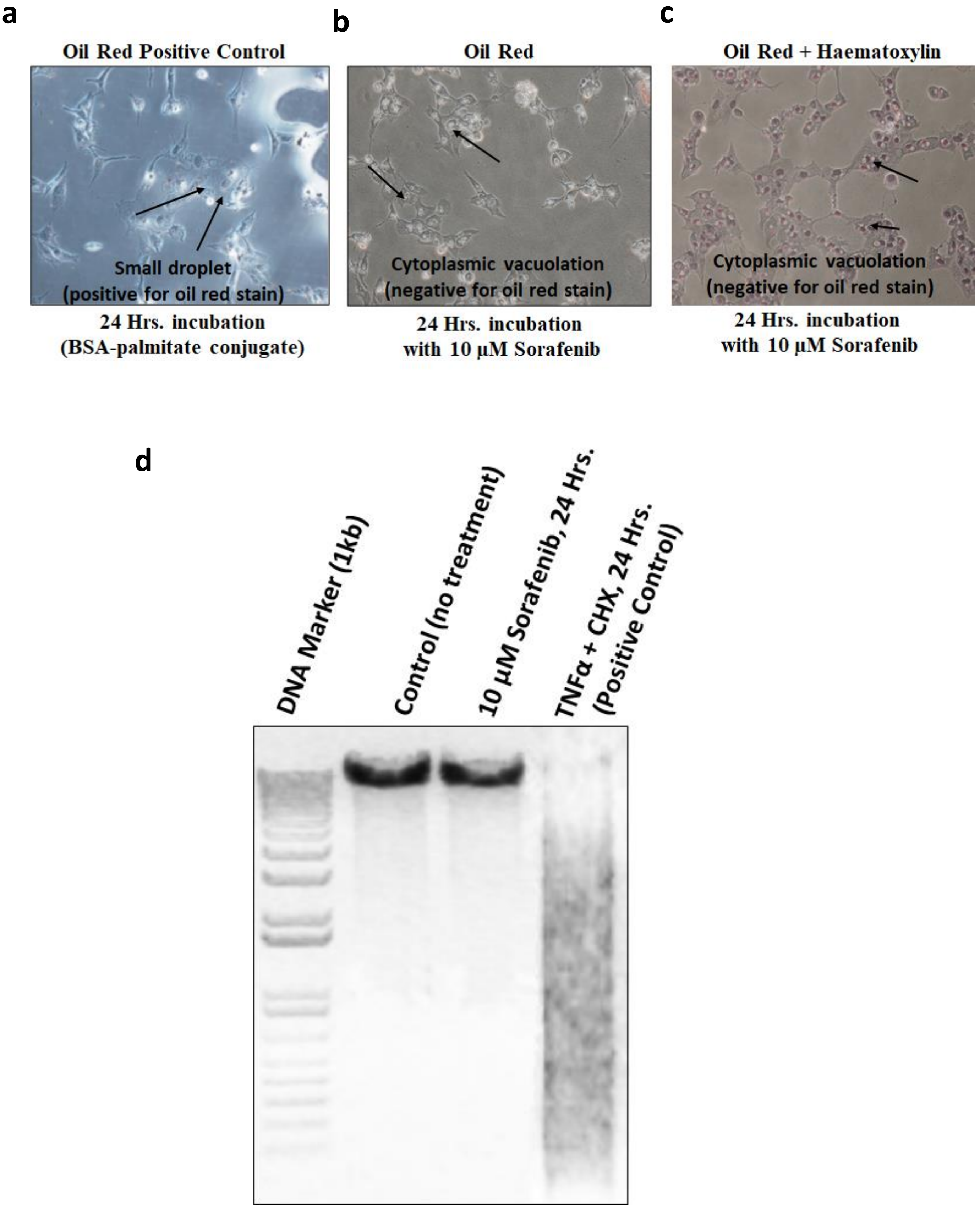
a
Oil Red Positive Control
Small droplet
(positive for oil red stain)
24 Hrs. incubation
(BSA-palmitate conjugate)
b
Oil Red
Cytoplasmic vacuolation
(negative for oil red stain)
24 Hrs. incubation
with 10 μM Sorafenib
c
Oil Red + Haematoxylin
Cytoplasmic vacuolation
(negative for oil red stain)
24 Hrs. incubation
with 10 μM Sorafenib
d
DNA Marker (1kb)
Control (no treatment)
10 μM Sorafenib, 24 Hrs.
TNFα + CHX, 24 Hrs.
(Positive Control)

Suppl. Fig. S3

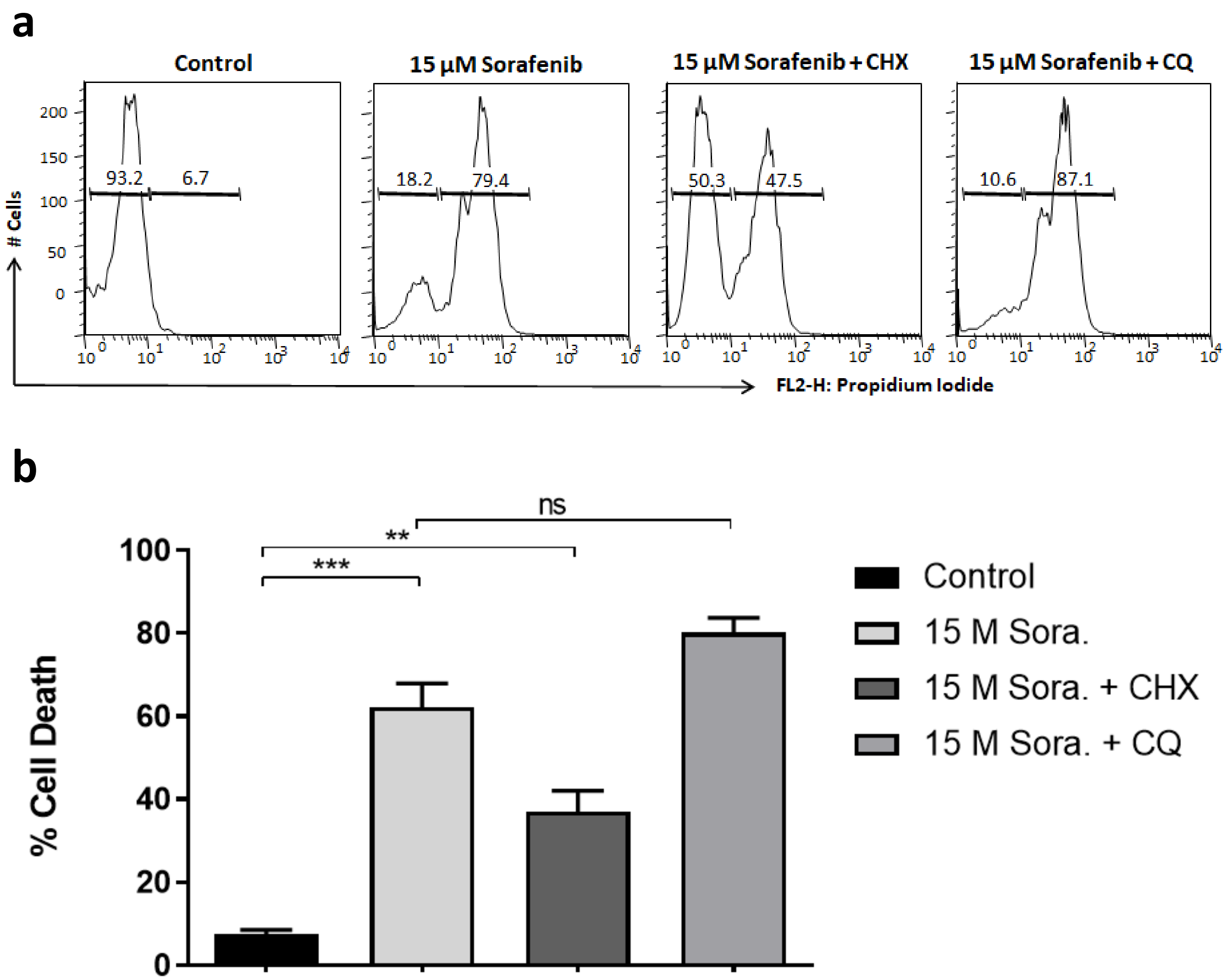

a
Control
15 µM Sorafenib
15 µM Sorafenib + CHX
15 µM Sorafenib + CQ
Cells
93.2
6.7
18.2
79.4
50.3
47.5
10.6
87.1
FL2-H: Propidium Iodide
b
% Cell Death
ns
**
***
Control
15 M Sora.
15 M Sora. + CHX
15 M Sora. + CQ

Suppl. Fig. S4

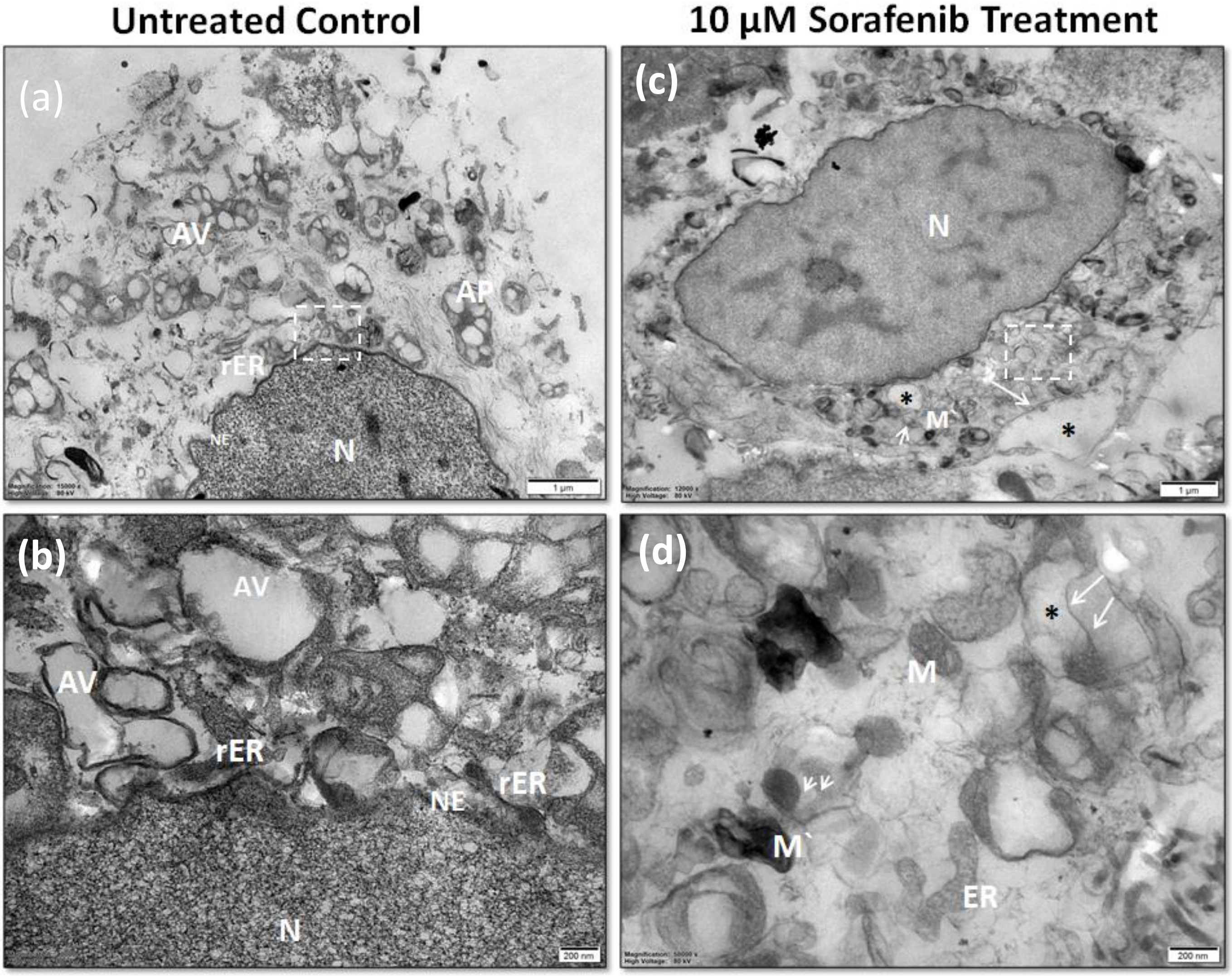

Untreated Control
10 µM Sorafenib Treatment
(a)
AV
AP
rER
NE
N
1 µm
(c)
N
M`
*
*
1 µm
(b)
AV
AV
rER
rER
NE
N
200 nm
(d)
M
*
M`
ER
200 nm

Suppl. Fig. S5

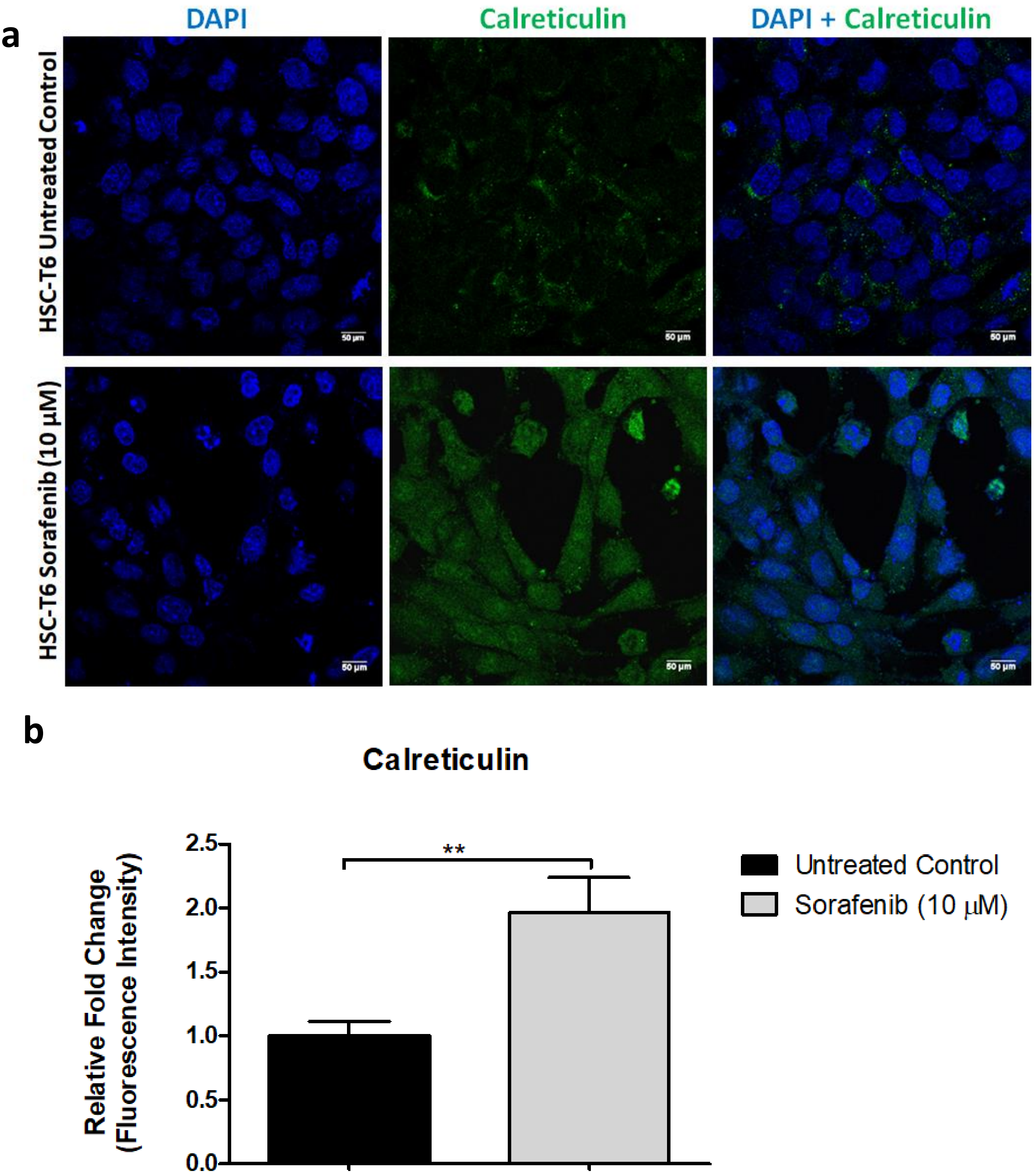
a
DAPI
Calreticulin
DAPI + Calreticulin
HSC-T6 Untreated Control
HSC-T6 Sorafenib (10 µM)
50 µm
b
Calreticulin
Relative Fold Change
(Fluorescence Intensity)
2.5
2.0
1.5
1.0
0.5
0.0
**
Untreated Control
Sorafenib (10 µM)

Suppl. Fig. S6

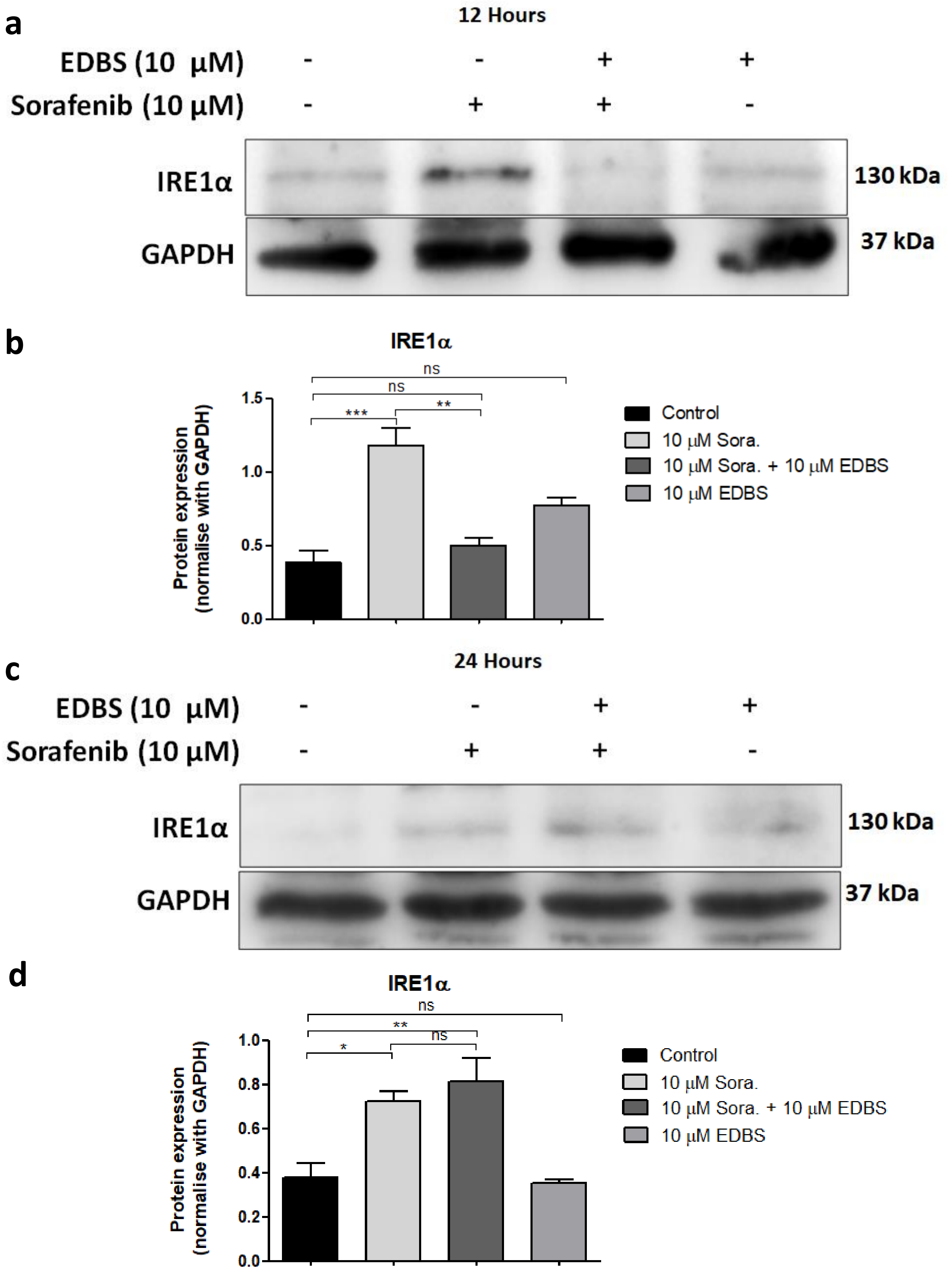

a
12 Hours
EDBS (10 μM) - - + +
Sorafenib (10 μM) - + + -
IRE1α
GAPDH
130 kDa
37 kDa
b
IRE1α
ns
ns
***
**
1.5
1.0
0.5
0.0
Protein expression (normalise with GAPDH)
Control
10 μM Sora.
10 μM Sora. + 10 μM EDBS
10 μM EDBS
c
24 Hours
EDBS (10 μM) - - + +
Sorafenib (10 μM) - + + -
IRE1α
GAPDH
130 kDa
37 kDa
d
IRE1α
ns
**
ns
*
1.0
0.8
0.6
0.4
0.2
0.0
Protein expression (normalise with GAPDH)
Control
10 μM Sora.
10 μM Sora. + 10 μM EDBS
10 μM EDBS

Suppl. Fig. S7

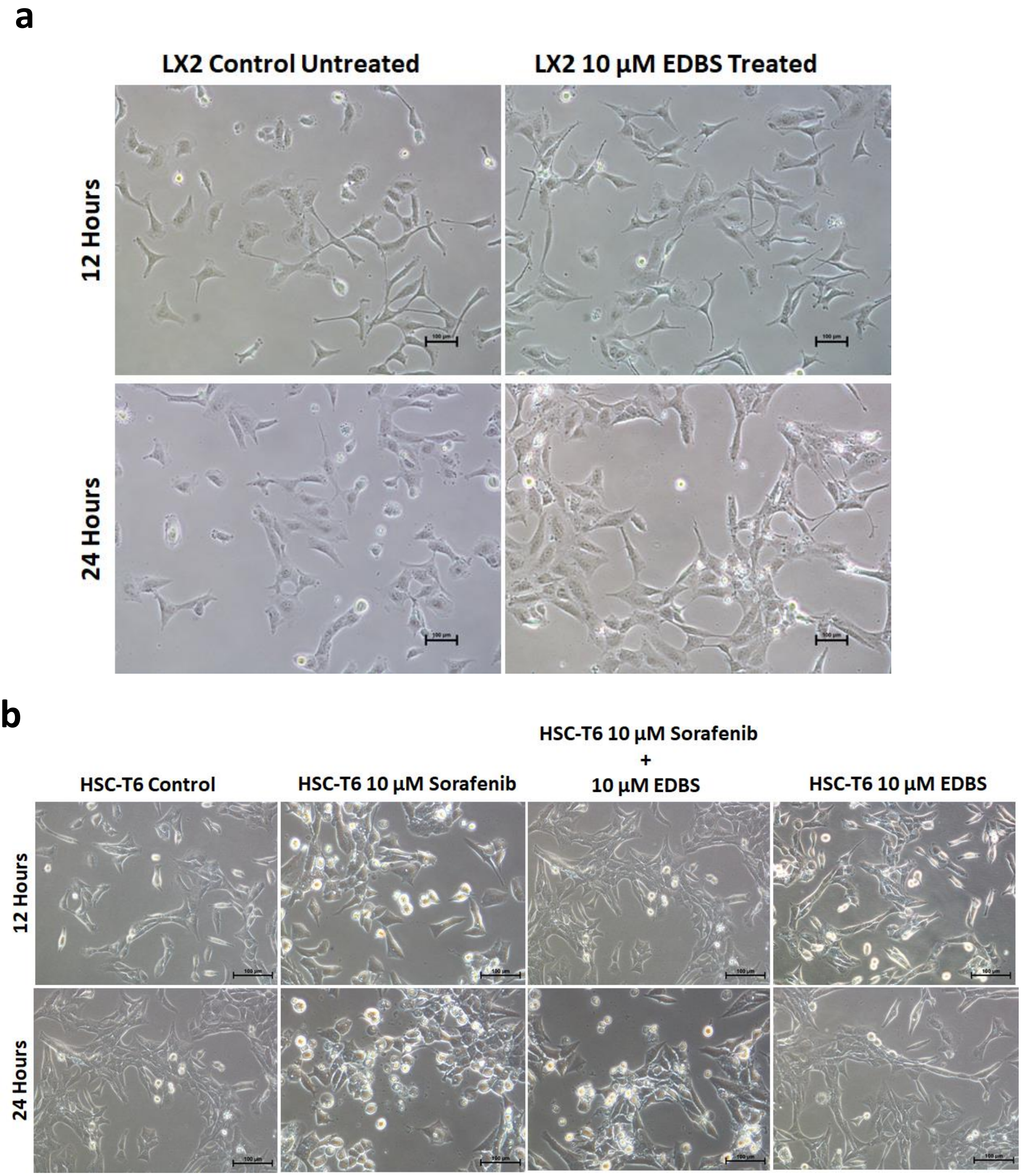
a
LX2 Control Untreated
LX2 10 µM EDBS Treated
12 Hours
24 Hours
b
HSC-T6 Control
HSC-T6 10 µM Sorafenib
HSC-T6 10 µM Sorafenib + 10 µM EDBS
HSC-T6 10 µM EDBS
12 Hours
24 Hours
